# Integrating Divergence-Based Proteomic Analysis and Directed Network Diffusion to Characterize Diagnosis-Anchored Molecular Variability at the Metabolic Syndrome–Migraine Interface

**DOI:** 10.3390/ijms27114820

**Published:** 2026-05-27

**Authors:** Bei Wang, Yulin Li, Yixing Liu, Dongran Han

**Affiliations:** 1 School of Life Sciences, Beijing University of Chinese Medicine, Beijing 102488, China; 202353009@bucm.edu.cn (B.W.); 20250931165@bucm.edu.cn (Y.L.); 2 School of Management, Beijing University of Chinese Medicine, Beijing 102488, China

**Keywords:** migraine, metabolic syndrome, plasma proteomics, proteomic variability, divergence-based proteomic analysis, directed network diffusion, network medicine

## Abstract

Metabolic syndrome (MetS) has been associated with migraine, but the prediagnostic phase and the molecular features preceding migraine onset remain unclear. We aimed to identify a diagnosis-anchored proteomic variability window and to characterize pathways, candidate bridge proteins, and druggable targets within a direction-consistent MetS-to-migraine molecular framework. We first assessed the association between baseline MetS and incident migraine in 452,471 UK Biobank participants using Cox models. We then conducted a nested proteomics case–control analysis stratified by MetS status and applied single-sample Jensen–Shannon divergence (sJSD), network proximity, directed diffusion, and drug–target proximity analyses. Baseline MetS was associated with a higher risk of incident migraine (hazard ratio 1.09, 95% confidence interval (CI) 1.01–1.18; *p* = 0.022). A diagnosis-anchored proteomic divergence pattern peaked 4.71–6.76 years before migraine diagnosis in the MetS stratum. The parallel within-stratum analysis in the NoMetS stratum showed no T2-centered peak. We identified 11 direction-consistent novel pathways, seven candidate bridge proteins spanning metabolic, endothelial, and brain tissues, and three candidate drugs. These findings support a diagnosis-stratified framework for studying MetS-related migraine and provide testable hypotheses for future mechanistic and pharmacological evaluation.

## 1. Introduction

Migraine is the second leading cause of disability worldwide [1], yet its molecular origins and pathways of susceptibility remain insufficiently understood [2]. Epidemiological evidence consistently demonstrates that metabolic syndrome (MetS) is a prevalent and clinically significant comorbidity in individuals with migraine, with cardiometabolic burden associated with a modest but consistently reported elevation in migraine risk [3,4,5]. However, this association is typically interpreted as a comorbid relationship arising from shared risk factors rather than as an interaction within a coupled biological system. As a result, traditional risk factor paradigms fail to explain how peripheral metabolic dysregulation might contribute to the neural vulnerability characteristic of migraine.

To move beyond traditional risk factor paradigms, a system biology perspective emphasizes that metabolic and neural circuits share a core functional imperative; both operate as predictive, interconnected networks designed to preserve homeostasis [2,6]. Metabolic networks anticipate and regulate energy demands, whereas neural circuits predict sensory threats and drive adaptive responses. Within this framework, MetS represents not only a cluster of metabolic abnormalities but also a state of heightened peripheral signal volatility, characterized by lipid-driven endothelial stress [7], inflammatory drift, and instability in energy-related signalling [6]. Conversely, migraine reflects a form of neural hypersensitivity in which cortical excitability and brainstem threat-prediction systems function near their stability margins [2]. This functional interdependence raises the possibility that chronic metabolic disturbances may increase the burden of peripheral signalling fluctuations on neural systems, thereby contributing to vulnerability in the transitions that manifest clinically as migraine attacks [8].

Building on this system-level view, multiomics studies have begun to identify shared genetic, lipidomic, and inflammatory mechanisms linking metabolic dysfunction with migraine [5,8]. For example, these studies have highlighted that in the context of cardiometabolic burden, migraine is accompanied by altered inflammatory signalling and dysregulated lipid metabolism [5]. However, most studies focus on patients with established disease or conceptualize risk as a static accumulation of cardiometabolic factors. Such approaches provide limited insight into the molecular features preceding migraine diagnosis and the pathways through which metabolic disturbances are relayed towards neural targets [9].

To address these unresolved diagnosis-stratified and pathway-level questions, divergence-based proteomic analysis provides a relevant quantitative basis. Theory predicts that systems approaching a state shift exhibit increasing variance, diminished resilience, and network reorganization, features that have been validated as early warning signatures [10] across complex disorders, including cancer metastasis, diabetic kidney disease, epilepsy, and depression [11,12,13]. Because these signatures manifest as quantifiable changes in molecular variability and network structure, they can be detected at scale in population-level proteomic distributions, thereby illuminating early molecular transitions that may link peripheral metabolic states to emerging neurological vulnerability. However, it remains unknown whether systematic proteomic divergence patterns can be detected within prediagnostic profiles linking MetS to incident migraine in large population cohorts.

**Figure 1:**
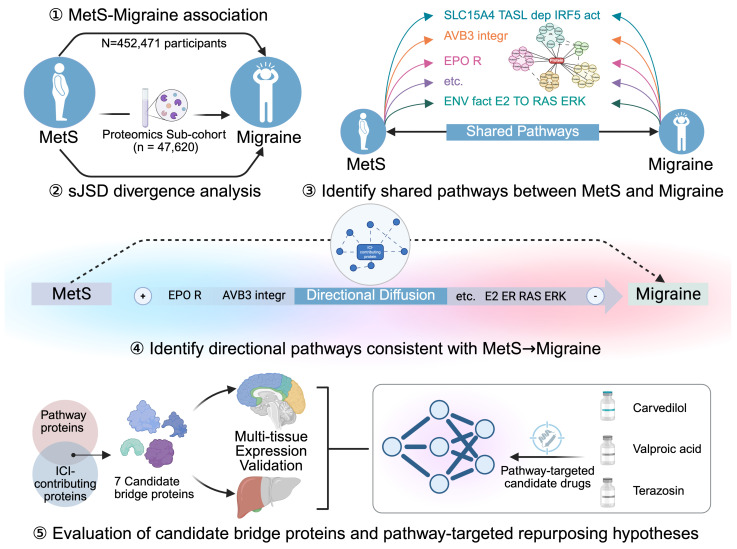
Study design and analytic framework. Prospective cohort participants were stratified by metabolic syndrome (MetS) status and monitored for incident migraine, including a nested proteomics subcohort with baseline plasma proteomic profiling (**➀**). single-sample Jensen–Shannon divergence (sJSD)-based divergence scores were computed across diagnosis-anchored windows to identify the most prominent window in this analysis (**➁**). Inconsistency index (ICI)-contributing proteins from this window were mapped onto curated MetS- and migraine-related pathway sets to identify shared pathways (**➂**). Directed diffusion (TieDIE) was applied to these shared pathways to quantify pathway-level direction consistency under a MetS-to-migraine source–target specification (**➃**). Genes from the 11 direction-consistent novel pathways identified in the directed diffusion analysis were intersected with the top 500 variability-associated proteins to identify seven candidate bridge proteins, which were evaluated using multitissue expression data and cross-referenced with drug–target databases to generate pathway-targeted repurposing hypotheses (**➄**). Created in BioRender. Wang, B. (2026) https://BioRender.com/c2dn1j7 (accessed on 21 May 2026).

This study therefore aimed to develop a diagnosis-stratified framework by characterizing proteomic variability before migraine in individuals with MetS and examining how this variability is organized across pathways, bridge proteins, and druggable targets. To achieve this goal, we leveraged time-stratified analyses of 47,620 UK Biobank participants with deep proteomic profiling (2923 proteins) to characterize the network features of cross-system proteomic variability (Figure 1). We identified a distinct diagnosis-anchored window of proteomic divergence peaking approximately five years before the diagnosis of migraine in individuals with MetS, a pattern not observed in the parallel within-stratum analysis of the NoMetS stratum. Through directed network diffusion modelling, we further revealed pathways exhibiting directional consistency with a MetS-to-migraine directional axis, and we identified candidate bridge proteins positioned at the interface of metabolic volatility and neural susceptibility. Together, these findings delineate cross-system proteomic variability at the MetS–migraine interface and provide a basis for mechanistic investigation of the identified variability window, pathways, and bridge proteins.

## 2. Results

### 2.1. MetS Is Associated with Incident Migraine

**Figure 2:**
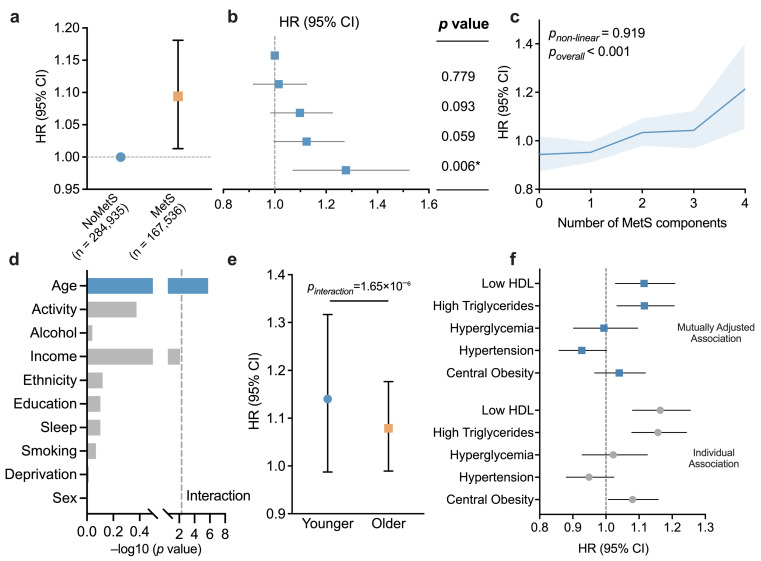
Prospective association between MetS and migraine risk. (**a**) Cox proportional hazards model for incident migraine according to baseline MetS status. (**b**) Hazard ratios stratified by component count; *p* values test linear trends. (**c**) Dose–response relationship; *p* values test nonlinearity and overall association. (**d**) Interaction *p* values (−$\log_{10}$ scale) across covariates. (**e**) Age-stratified models; $p_{\mathrm{interaction}}$ tests for age modification (unadjusted; Bonferroni-adjusted *p* = 1.65 × $10^{-5}$). (**f**) Hazard ratios for individual components in mutually adjusted versus individual models. Error bars and shaded bands represent 95% confidence intervals. The asterisk in panel (**b**) denotes statistical significance. In panels (**a**,**b**,**f**), dashed lines mark the null HR value of 1; in panel (**d**), the dashed line marks the Bonferroni-corrected significance threshold for interaction tests.

To assess whether MetS and its components are prospectively associated with incident migraine, we first analyzed the UK Biobank cohort using Cox proportional hazards models and complementary dose–response and component-specific analyses (Figure 2). The Cox analysis cohort included 452,471 participants (Supplementary Table S1). During a median follow-up of 13.71 years (interquartile range 12.96–14.44 years), 4283 incident migraine cases were identified. MetS was associated with a higher risk of incident migraine. This association remained significant even after adjusting for age, sex, ethnicity, socioeconomic status, and lifestyle factors (Figure 2a; HR 1.09, 95% CI 1.01–1.18; *p* = 0.022).

The risk of migraine increased with the number of abnormal MetS components beyond central obesity, with hazard ratios of 1.00, 1.02, 1.10, 1.12, and 1.28 for 0 to 4 components, respectively (linear trend *p* < 0.001; Figure 2b,c). The dose–response curve indicated no nonlinearity (*p* = 0.919), supporting a robust dose-dependent relationship. The association differed by age (Bonferroni-adjusted interaction *p* = 1.65 × $10^{-5}$; Supplementary Table S2), but the stratified estimates remained consistent across age groups (Figure 2d,e and Supplementary Figure S1).

Individual component analyses revealed significant associations of low HDL-C levels, elevated triglyceride levels, and central obesity with migraine risk. In models adjusted for all MetS components (Figure 2f), low HDL-C and elevated triglycerides were independently linked to increased migraine risk (HDL-C: HR 1.12, 95% CI 1.03–1.21; *p* = 0.008; triglycerides: HR 1.12, 95% CI 1.03–1.21; *p* = 0.005). Central obesity (HR 1.04, 95% CI 0.97–1.12; *p* = 0.302), hypertension (HR 0.93, 95% CI 0.86–1.00; *p* = 0.055), and hyperglycaemia (HR 0.99, 95% CI 0.90–1.10; *p* = 0.908) were not significantly associated. Taken together, these findings indicate that MetS is modestly associated with incident migraine and that this association largely reflects stronger associations for dyslipidaemic components, particularly low HDL-C and elevated triglycerides.

### 2.2. Proteomic Divergence Peaks at T2 in the MetS Stratum

**Figure 3:**
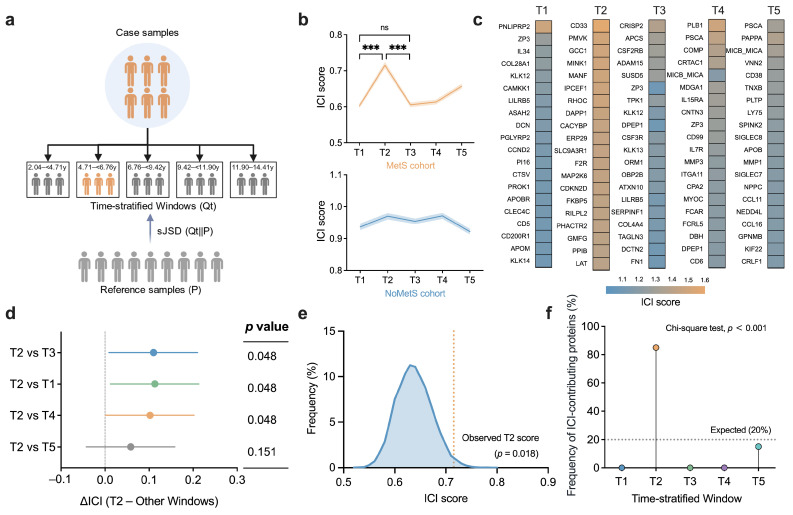
Migraine diagnosis-anchored proteomic variability. (**a**) Case samples partitioned into five prediagnostic windows (T1–T5, 2.04–14.41 years); sJSD compared window proteome distributions ($Q_{t}$) with those of baseline controls (*P*). Orange icons denote case samples and the highlighted case window, whereas grey icons denote reference samples and the other windows. (**b**) ICI trends shown separately for the MetS and NoMetS strata; asterisks denote pairwise significance for comparisons between T2 and the other time windows within the MetS stratum; the NoMetS stratum values are shown as a parallel within-stratum reference. Orange and blue lines represent the MetS and NoMetS strata, respectively; *** denotes adjusted *p* < 0.001 and ns denotes not significant in Tukey’s multiple comparisons following repeated-measures one-way ANOVA. (**c**) Heatmap of the top 20 ICI-contributing proteins ranked independently within each diagnosis-anchored window in the MetS cohort. Within each column, rows list the top 20 proteins for the corresponding window T1–T5; protein lists therefore differ across columns. Cell colour encodes the protein-level ICI score (cumulative protein-level sJSD contribution for that single protein) at the corresponding window, with lower values shown in blue and higher values in orange (see colour bar). (**d**) ΔICI between T2 and other windows; labels indicate BH-adjusted *p* values from pairwise permutation tests. Colours distinguish the T2-versus-window comparisons; the grey point denotes the non-significant comparison, and the vertical dashed line marks ΔICI = 0. (**e**) Observed T2 score compared with the resampling-based null distribution (10,000 iterations). The blue density indicates the null distribution and the orange dashed line marks the observed T2 score. (**f**) Proportion of ICI-contributing proteins per window; the chi-square test evaluated departure from uniform. The orange point highlights T2, other colours distinguish the remaining time windows, and the horizontal dashed line marks the expected 20% frequency under a uniform distribution. Panel (**a**) was created in BioRender. Wang, B. (2026) https://BioRender.com/75dzmj2 (accessed on 11 May 2026).

To identify a window of proteomic divergence before migraine diagnosis, we applied the sJSD framework to analyze 2923 proteins across five diagnosis-anchored windows (T1–T5, spanning 2.04–14.41 years before diagnosis; Figure 3a). This analysis included 28 incident migraine cases per diagnosis-anchored window in the MetS group and 42 per window in the NoMetS group. We observed a pronounced ICI peak in the MetS cohort at the T2 window (4.71–6.76 years). The ICI at T2 was 0.715, indicating an 18.8% increase from T1 (0.602) and an 18.2% increase from T3 (0.605). Even compared with the more distant T5 window (0.657), T2 was 8.8% greater (Figure 3b, upper panel; Supplementary Table S3). In contrast, T1 and T3 had nearly identical values (0.602 vs. 0.605). The NoMetS cohort showed variation across windows, with no clearly distinct T2 peak (Figure 3b, lower panel). At the individual protein level, the top 20 ICI contributors at T2 showed larger amplitude fluctuations than those in adjacent windows did (Figure 3c).

Pairwise permutation tests with Benjamini–Hochberg correction revealed that T2 ICI values were significantly greater than those of T1, T3, and T4 (all *q* < 0.05). However, the difference from T5 (8.8%) was not significant (*q* = 0.151) (Figure 3d and Supplementary Table S4). To rule out the possibility that the observed T2 peak could have occurred by chance, permutation-based resampling with 10,000 iterations revealed that the observed T2 value exceeded 98.2% of the null distribution (*p* = 0.018; Figure 3e and Supplementary Table S5). Bootstrap resampling with 1000 iterations demonstrated that the T2-minus-adjacent-window differences remained positive in 98.5% or more of bootstrap iterations, indicating stability (Supplementary Table S6). In parallel, peak frequency analysis of ICI-contributing proteins across windows revealed an overrepresentation at T2 relative to a uniform distribution ($\chi^{2}$ goodness-of-fit: $\chi^{2}$ = 2725.00, df = 4, *p* < 0.001; Figure 3f). Sensitivity analyses conducted under alternative parameter settings and cohort specifications consistently reidentified T2 as the most prominent divergence window (Supplementary Table S7). To show the participant-level values underlying these window-level ICI estimates, individual-level ICI distributions and their numerical summaries are provided in Supplementary Figure S2 and Supplementary Table S8, respectively. This distributional analysis shows a right-shifted upper tail at T2 in the MetS group. The parallel within-stratum analysis in the NoMetS stratum showed no T2-centered peak. Additional robustness analyses showed that the T2 peak remained stable in 27 of 28 targeted leave-one-out iterations and across all six prespecified top-protein cutoffs (Supplementary Tables S9 and S10). Together, these analyses identify a T2-centered proteomic-profile divergence pattern in the MetS stratum.

### 2.3. Shared Pathways Link T2-Window Proteomic Divergence to MetS–Migraine Comorbidity

**Figure 4:**
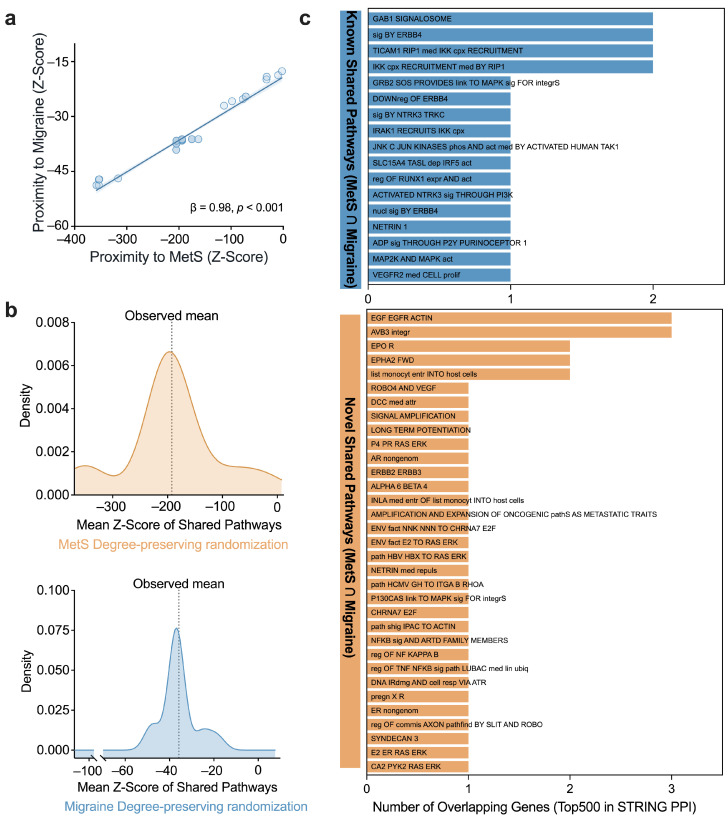
Shared network pathways jointly proximal to MetS and migraine and intersecting T2 variability-associated proteins. (**a**) Shortest-path proximity *Z* scores for 50 shared pathways to the MetS (*x*-axis) and migraine (*y*-axis) modules; the regression line shows the correlation. (**b**) Degree-preserving randomizations generated null distributions for mean pathway proximity (top: MetS, bottom: Migraine); dashed lines mark observed values. (**c**) Fifty shared pathways ranked by overlapping genes with the top 500 T2 variability-associated proteins; blue indicates known pathways and orange indicates novel pathways.

To examine whether the T2-centered proteomic divergence observed in the MetS group is embedded in pathways jointly implicated in MetS and migraine, we next performed network proximity analysis between disease-associated genes and curated pathways and mapped these pathways onto the proteins contributing to the T2-window divergence signal (Figure 4). Network proximity analysis revealed 50 pathways significantly linked to both MetS (107 mapped genes) and migraine (56 mapped genes). The proximity patterns between MetS and migraine were strongly correlated (β = 0.98; *p* < 0.001; Figure 4a), with mean *z* scores for both diseases far exceeding null expectations (MetS *z* = −192.41; Migraine *z* = −35.83; Figure 4b). All 50 pathways exhibited direct network connectivity to the top 500 proteins from the T2 window, where 41 pathways (82%) overlapped with 1 protein, 7 (14%) with 2 proteins, and 2 (4%) with 3 proteins (Figure 4c).

Literature-based analysis categorized pathways into 17 known and 33 novel groups, with “Novel” denoting pathways that lacked prior pathway-level associations in our structured literature review. The known pathways had established links to MetS or migraine and were primarily associated with innate immune signalling, such as IRAK1/IKK, TICAM1/RIP1 recruitment, and JNK activation, as well as receptor tyrosine kinase pathways such as NTRK3, ERBB4, and VEGFR2. In contrast, the novel pathways, despite containing disease-associated genes, lacked previous pathway-level associations. They included nuclear factor kappa-light-chain-enhancer of activated B cells (NF-κB) regulatory mechanisms, DNA damage response pathways, neuroplasticity and axon-guidance processes, nongenomic hormone signalling, and integrin-mediated adhesion (Supplementary Table S11). Directed signal-propagation analysis was then conducted on this shared pathway set, with a particular focus on the 33 novel pathways. These findings indicated that the proteomic divergence observed at T2 was organized within a discrete set of pathways that were jointly proximal to both MetS and migraine modules, encompassing a substantial fraction of mechanistically plausible yet previously underrecognized bridge pathways.

### 2.4. Direction-Consistent Pathways Link MetS and Migraine in Directed Diffusion Analysis

**Figure 5:**
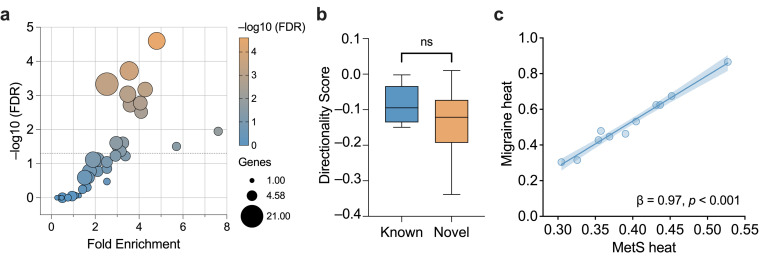
Directed diffusion and pathway direction consistency. (**a**) Sixteen pathways enriched in the MetS–migraine mediator subnetwork (false discovery rate [FDR] < 0.05); the bubble size indicates the mediator gene count. (**b**) Directionality scores for known versus novel pathways; *p* value from the Mann–Whitney U test. ns, not significant. (**c**) Linear correlation between MetS-seeded and migraine-seeded diffusion heat for 11 novel pathways.

To assess whether the shared pathways between MetS and migraine occupy directionally consistent positions along a MetS-to-migraine diffusion axis, we applied directed diffusion analysis to the mediator subnetwork connecting disease-associated genes (Figure 5). Directed diffusion analysis revealed 16 pathways significantly enriched in mediator subnetworks connecting MetS and migraine genes (FDR < 0.05; Figure 5a). Among these pathways, 11 were novel, and 5 were known pathways. The novel pathways with the greatest enrichment in terms of folds were LUBAC (linear ubiquitin chain assembly complex)-mediated TNF–NF-κB regulation (fold = 7.60; FDR = 0.011), an environmental E2-to–RAS–ERK signalling pathway (fold = 4.09; FDR = 0.0031), and p130Cas-to-integrin MAPK signalling (fold = 4.06; FDR = 0.0017). Across all 16 enriched pathways, fold enrichment ranged from 2.54 to 7.60, with a median of 3.58, spanning inflammatory cascades, integrin adhesion, receptor signalling, and calcium-dependent mechanisms (Supplementary Table S12).

Directionality scores, which describe the relative axial position of each pathway between the MetS and migraine modules in the directed diffusion framework, were not significantly different between the novel and known pathways (Mann-Whitney U test *p* = 0.53; Figure 5b). This finding suggests that the novel pathways showed a directionality pattern comparable to that of established mechanisms. Among the 11 novel pathways, forwards and backwards diffusion heat were strongly linearly correlated (β = 0.97, *p* < 0.001; Figure 5c), indicating the presence of shared molecular substrates between MetS and migraine. Together, these results identified a subset of shared pathways linking the MetS and migraine modules in the directed diffusion analysis and provided a focused substrate for the identification of molecular bridge proteins.

### 2.5. T2-Associated Bridge Proteins Support Pharmacological Hypotheses

**Figure 6:**
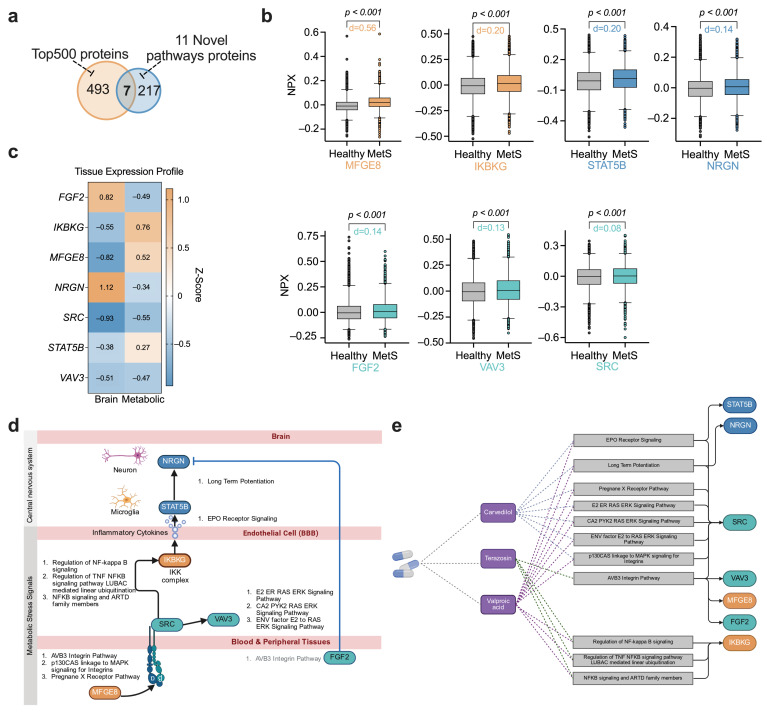
Bridge proteins and pharmacological context. (**a**) Intersection of the top 500 T2 variability-associated proteins and 224 unique genes represented across 11 novel pathways identified 7 candidate bridge proteins. (**b**) Baseline plasma NPX distributions in healthy controls versus the MetS-only group; labels report Cohen’s *d* and unadjusted *p* values (BH-adjusted *q* values are reported in Supplementary Table S13). (**c**) Tissue expression *z* scores across brain-enriched and metabolically enriched tissues. (**d**) Schematic representation of 7 proteins across metabolic tissues, endothelium/blood–brain barrier (BBB), and the central nervous system (CNS); pathway annotations represent literature-supported hypotheses linking metabolic instability to neural vulnerability. (**e**) Network linking PxEA-prioritized drugs to 11 novel pathways and 7 bridge proteins (FDR < 0.05); the three drugs directly targeting at least one of the 11 novel pathways are highlighted. Panel (**d**) was created in BioRender. Wang, B. (2026) https://BioRender.com/lcgoi27 (accessed on 11 May 2026). Panel (**e**) was created in BioRender. Wang, B. (2026) https://BioRender.com/oo8820i (accessed on 11 May 2026).

To prioritize candidate molecular bridges between MetS and migraine and to explore their pharmacological context, we integrated T2-window proteomic variability with pathway, tissue expression and drug–target proximity information (Figure 6). Intersection analysis was performed between the 224 unique genes represented across the 11 direction-consistent novel pathways and the top 500 variability-associated proteins identified by sJSD analysis during the 4.71–6.76-year window. This revealed seven candidate bridge proteins, SRC, IKBKG, FGF2, MFGE8, VAV3, NRGN, and STAT5B (Figure 6a). These proteins belong to the 11 direction-consistent novel pathways and showed T2-window sJSD contributions, comprising 3.1% of the novel pathway genes and 1.4% of the top 500 proteins. The convergence of pathway consistency and T2-window proteomic variability across these seven bridge proteins is consistent with their mechanistic plausibility within the proposed MetS–migraine direction-consistent network pattern. This convergence is reflected by their participation in the 11 direction-consistent novel pathways, elevated NPX levels in MetS, and contrasting brain versus metabolic tissue expression profiles (Supplementary Table S13).

In the UK Biobank Olink plasma proteomics cohort, comparisons restricted to participants without baseline migraine revealed elevated NPX levels of all seven bridge proteins in the MetS-only group (*n* = 17,739) versus healthy controls (*n* = 29,420). These differences remained significant after Benjamini–Hochberg correction (all *q* < 0.05; Supplementary Table S13; Figure 6b). Effect sizes, expressed as Cohen’s *d*, varied substantially: MFGE8 had the greatest effect (*d* = 0.56), followed by IKBKG (*d* = 0.20) and STAT5B (*d* = 0.20). FGF2 (*d* = 0.14), NRGN (*d* = 0.14), VAV3 (*d* = 0.13), and SRC (*d* = 0.08) exhibited moderate to small effects. Tissue-level expression analysis revealed pronounced spatial heterogeneity among the seven bridge proteins between brain and metabolic tissues (Figure 6c). FGF2 was enriched in brain tissues, with a mean *z* score of 0.82. In contrast, IKBKG and MFGE8 were more abundant in metabolic-related tissues, with *z* scores of 0.76 and 0.52, respectively. SRC and VAV3 showed no clear tissue-specific enrichment and presented a relatively balanced distribution across the examined tissues.

PPI networks combined with tissue expression data facilitated the functional localization of seven bridge proteins across anatomical compartments (Figure 6d). SRC participated in six novel pathways, including three RAS–ERK signalling pathways, one integrin MAPK signalling route, Epo receptor signalling, and one nuclear receptor pathway, and acted as a central signalling hub. MFGE8, FGF2, and VAV3 converged on the $\alpha_{v}\beta_{3}$ integrin pathway. IKBKG participated in three NF-κB–related novel pathways (regulation of NF-κB signalling, TNF–LUBAC–NF-κB, and NF-κB signalling with ARTD family members), NRGN is associated with long-term potentiation, and STAT5B is involved in Epo receptor signalling (Supplementary Table S13). The spatial distribution of these proteins across brain tissues, the circulatory system, and metabolic organs highlights their role as cross-system bridging molecules.

PxEA was used to evaluate the topological proximity between drug targets and 11 novel pathway genes within the PPI network. Among the drugs indicated for MetS or migraine, five candidates were significantly enriched (FDR < 0.05); among them, three directly targeted at least one of the 11 direction-consistent novel pathways and are highlighted in Figure 6e (carvedilol, valproic acid, and terazosin). Carvedilol, a MetS medication, had the highest enrichment score (ES = 104.4), targeting 7 of the 11 pathways, whereas valproic acid, used for migraines, targeted 10 pathways (ES = 101.1), and terazosin, an antihypertensive used in MetS-related indications, targeted 4 pathways (ES = 75.1) (Supplementary Table S14), generating repurposing hypotheses for future evaluation based on their shared pathway targeting. Collectively, these analyses prioritize a small set of bridge proteins supported by T2-window proteomic divergence and direction-consistent pathways at the interface between metabolic dysregulation and migraine susceptibility and highlight existing pharmacological agents whose targets overlap these cross-system signalling routes as projected drug repurposing hypotheses for future mechanistic and pharmacological evaluation.

## 3. Discussion

To address the limited understanding of how metabolic dysregulation might relate to migraine susceptibility over time, we integrated prospective cohort data with baseline proteomics stratified by time-to-migraine diagnosis and directed diffusion-based network modelling to explore when, and through which molecular pathways, MetS-related perturbations might be associated with increased migraine risk. Our study reframes the MetS–migraine comorbidity by integrating time-stratified proteomics with directed network dynamics, characterizing the relationship not as a static accumulation of risk but as a dynamic cross-system vulnerability pattern. We identify a proteomic divergence pattern peaking 4.71–6.76 years before migraine onset within a convergent T2 window in the MetS group. In the NoMetS stratum, the parallel within-stratum analysis showed no T2-centered peak. This proteomic divergence pattern is structurally underpinned by a directionally consistent subnetwork comprising 11 novel pathways alongside established routes, which intersects with the T2-window divergence signal to prioritize seven candidate bridge proteins (e.g., MFGE8, SRC, NRGN) whose tissue-expression profiles place them at the interface of metabolic, endothelial, and neural tissues. Collectively, these data suggest that MetS may act as a persistent source of peripheral signal volatility rather than a static risk factor, with this variability mapped to these molecular relays, thereby supporting a framework for understanding how chronic metabolic noise may be associated with reduced resilience of neural systems.

Against this system-level backdrop, we next examined how individual MetS components related to migraine risk at the epidemiologic level. Epidemiologically, the association between MetS and migraine was unevenly distributed across the syndrome components. Cox regression confirmed an overall link (HR 1.09, 95% CI 1.01–1.18). However, in the mutually adjusted models, only low HDL-C levels (HR 1.12, *p* = 0.008) and elevated triglyceride levels (HR 1.12, *p* = 0.005) retained independent associations with incident migraine; central obesity, hypertension, and hyperglycaemia did not reach significance. This selective concentration of risk within the lipid domain aligns with earlier reports implicating HDL dysfunction [3] and triglyceride-rich lipoproteins [5] in migraine and vascular dysfunction. Mechanistically, chronic dyslipidaemia produces oxidized lipoproteins and bioactive ceramides that activate endothelial cells and sustain low-grade vascular inflammation [14,15]. Because these bioactive species are continually generated and cleared [14,15], circulating lipids impose recurrent signalling bursts rather than the relatively constant mechanical load of adiposity. Notably, our observation that independent associations were confined to lipid components rather than adiposity measures aligns with this mechanistic distinction. Within our system framework, this pattern suggests that the MetS–migraine link is preferentially loaded onto dynamic lipid signalling rather than static anthropometric burden, which is consistent with the hypothesis that migraine susceptibility may be more responsive to chronic signalling noise than to structural pressure alone. While these epidemiologic associations do not establish causality, they provide a population-level premise for the proteomic divergence we subsequently observed in the T2 window. Together, these observations are consistent with lipid-driven systemic perturbations, rather than adiposity alone, representing one plausible epidemiologic substrate for the T2-window proteomic divergence observed in this analysis and suggesting this axis as a priority for future investigation.

At the T2 window, the convergence of the peak ICI and heightened protein fluctuation indicates pronounced proteomic-profile divergence [16]. These findings suggest that the epidemiological risk observed at the population level is accompanied by elevated proteomic-profile divergence in this diagnosis-anchored window [11,16]. Similar divergence-based molecular patterns have also been discussed in studies of cancer and viral infections [12,16,17]. In the NoMetS stratum, the parallel within-stratum analysis showed variation across windows with no clearly distinct T2 peak. Consequently, T2 represents a diagnosis-anchored window of elevated divergence in proteomic profiles in the MetS group, providing a focused basis for dissecting the specific molecular pathways potentially linking metabolic disturbances to neural susceptibility [11,12,16].

Within this vulnerability window, the set of pathways identified by network modelling exhibited MetS-to-migraine directional consistency. Specifically, the recovery of NF-κB–mediated inflammatory signalling and VEGF-related vascular pathways [18,19] provides an internal plausibility check, indicating that the directed diffusion framework (TieDIE) [20] can recover pathways with prior pathway-level relevance to metabolic and vascular dysfunction and migraine. This pattern supports the interpretability of the network ranking and reduces concern that the findings are driven solely by topological artefacts. Beyond these known axes, the 11 novel pathways further refine the cross-system interface. Pathways involving LUBAC-mediated linear ubiquitination and NF-κB regulatory mechanisms [21,22] suggest potential amplification of systemic stress; integrin adhesion and receptor tyrosine kinase modules [23,24] suggest complex endothelial sensing and barrier gating; and oestrogen-responsive neuroplasticity pathways [25] connect this network pattern to neural circuits implicated in female-predominant migraine susceptibility. Collectively, these patterns outline a metabolically anchored, endothelial-facing, and neuronally connected subnetwork compatible with a cross-system vulnerability architecture rather than diffuse, nonspecific coupling. Although hub-driven effects cannot be fully excluded, the persistence of this topology under degree-preserving randomization suggests a non-random, direction-consistent subnetwork pattern within the cross-system vulnerability architecture suggested by the epidemiologic and proteomic divergence findings.

At the structural intersection between ICI-contributing proteins from the T2 window and the identified directed subnetwork, seven proteins emerged as candidate bridge nodes. Tissue specificity analysis revealed that these proteins are not uniformly distributed but are distinctly enriched in peripheral, endothelial, and neural tissues. Their known functions align with this anatomical segregation. In peripheral tissues, MFGE8 and IKBKG regulate lipid uptake and inflammatory signalling [22,26], suggesting that they sense systemic metabolic fluctuations. In vascular-enriched tissues, SRC [27] and FGF2 [28] have opposing effects on endothelial integrity: SRC promotes barrier disassembly, while FGF2 supports barrier stabilization. The concurrent identification of these countervailing factors suggests that the high variance observed in T2 may reflect a molecular “tug-of-war” and a state of homeostatic strain at the vascular interface. Finally, in the central nervous system, NRGN and STAT5B modulate synaptic plasticity and excitability. Taken together, these anatomically distinct but functionally connected nodes are compatible with a hypothesized cross-system network pattern and are consistent with a conceptual link from metabolic noise to neural susceptibility with endothelial involvement highlighted by the network and tissue-expression analyses.

In the exploratory pathway-based drug–target proximity analysis, three previously approved agents, namely, valproic acid, carvedilol and terazosin, exhibited the strongest alignment with the novel pathway set. Valproic acid, a guideline-recommended prophylactic for migraine [29], was enriched across ten pathways. Beyond its GABAergic and sodium-channel actions, valproic acid reportedly reduces endothelial ICAM-1 and VCAM-1 expression via NF-κB inhibition, which is consistent with the inflammatory–endothelial axis highlighted here. Carvedilol, a vasodilating β-blocker widely used for MetS-related hypertension, was enriched across seven pathways; its antioxidant activity has been linked to improved endothelium-dependent vasodilatation [30], and β-blockers have demonstrated migraine-preventive efficacy in randomized trials, aligning with the metabolic–vascular tier of the proposed network pattern. Terazosin, an $\alpha_{1}$-adrenergic antagonist prescribed for MetS-related conditions, was enriched across four pathways; although it has not previously been implicated in migraine, it activates Pgk1 and confers neuroprotection in rodent stroke and sepsis models [31], which may be relevant to the neuronal vulnerability emphasized in our framework. Taken together, these convergent signals provide biologically coherent repurposing hypotheses that may help prioritize future pharmacoepidemiologic and, where feasible, experimental or clinical evaluation in MetS-related contexts.

This study has several limitations. The observational design precluded definitive causal inference, and the directionality suggested by diffusion-based modelling reflected direction-consistent patterns under a directed diffusion framework rather than proven biological flow. Because proteomic measurements were obtained only at baseline and the timing of MetS onset was unavailable, we could not determine whether the diagnosis-anchored T2 window corresponds to a clinically forward-looking transition point relative to MetS duration. The Olink panel, although comprehensive, is enriched for cardiometabolic and inflammatory proteins and may underrepresent neuron-specific markers. Additionally, NPX is Olink’s arbitrary log_2_-scaled unit and supports relative quantification only; measurements for low-abundance analytes near or below the limit of detection may have reduced reliability. Although batch- and plate-related variation was minimized through UKB-PPP quality-control procedures, technical variation inherent to proximity extension assays cannot be fully excluded, and imputation of missing protein values may introduce additional measurement uncertainty. Medication use was represented by baseline treatment categories, which may have contributed to residual misclassification of MetS components. Replication in independent cohorts with denser temporal sampling and broader ancestral representation is needed. Finally, although these projected drug repurposing signals were biologically coherent, their clinical relevance in individuals with MetS requires experimental and pharmacoepidemiologic validation.

## 4. Materials and Methods

### 4.1. Study Participants

The UK Biobank included approximately 500,000 adults aged 40–69 years who were enrolled between 2006 and 2010 [32]. To examine incident migraine epidemiologically, we excluded individuals who had migraine at baseline and applied a two-year landmark period [33] to reduce the likelihood of reverse causation; this resulted in a cohort of 452,471 participants. For proteomic analyses, we restricted the sample to UK Biobank participants with baseline Olink Explore 3072 proteomic data (*n* = 53,013). After successful linkage was established with the curated MetS and migraine labels, the final proteomic subset comprised 47,620 participants.

### 4.2. Proteomic Profiling

Plasma proteins were assessed using the Olink Explore 3072 platform, which employs proximity extension assays (PEA) across various panels, including cardiometabolic, inflammatory, neurologic, and oncologic. Protein levels are reported as normalized protein expression (NPX) values on a log_2_ scale. The dataset, which included 2923 distinct protein targets, was derived from the QC-processed UK Biobank Pharma Proteomics Project (UKB-PPP) NPX dataset and used for further analysis [34,35]. These PEA-based protein profiles provide high-specificity, multiplex proteomic measurements at population scale, supporting analyses of diagnosis-anchored proteomic divergence and pathway-level molecular context in this cohort.

### 4.3. Exposure and Outcome Definitions

MetS was defined using the 2005 International Diabetes Federation (IDF) criteria [36]. Participants met the MetS classification if they had central obesity, identified by a waist circumference of ≥94 cm for men or ≥80 cm for women, plus at least two of the following factors: elevated triglycerides (≥1.7 mmol/L); low high-density lipoprotein (HDL) cholesterol (<1.03 mmol/L for men or <1.29 mmol/L for women); elevated blood pressure (systolic ≥ 130 mmHg or diastolic ≥ 85 mmHg); and elevated glucose (≥5.6 mmol/L), with baseline lipid-lowering, antihypertensive, or insulin medication use counted toward the corresponding component.

Migraine diagnoses were determined from linked health records with the International Classification of Diseases, 10th Revision (ICD-10) code G43, supplemented by self-reported migraine history. At baseline, participants reported whether they had ever had a migraine (past or current) and, where applicable, the date of their migraine diagnosis. These self-reported responses were combined with ICD-10 G43 records to define prevalent and incident migraine. Prevalent migraine at baseline was identified by an ICD-10 G43 record dated on or before baseline, self-reported migraine at baseline, or a self-reported diagnosis prior to baseline. Incident migraine was defined as the first occurrence of either ICD-10 G43 coding or self-reported migraine after baseline. Follow-up duration was measured from the baseline assessment date to the earliest incident of migraine, death, or administrative censoring (30 November 2022).

### 4.4. Missing Data Imputation

Covariate missingness included item non-response (e.g., “do not know” or “prefer not to answer”) and incomplete questionnaire or assessment entries in the UK Biobank baseline data. Missing covariate data, including MetS components and adjustment variables, were imputed in R using a published random forest strategy with predictive mean matching [34], implemented with the missRanger package. Five imputed datasets were generated; each used ten iterations and 200 trees. Responses recorded as “do not know” were treated as missing and were imputed, whereas “prefer not to answer” responses were set to missing after imputation. Age, sex, and outcome/time variables were not included. Before imputation, patterns of covariate missingness were summarized by variable and evaluated for associations with age and sex to inform missing-at-random assumptions, and out-of-bag prediction errors from the missRanger models were summarized across imputations as a measure of imputation model performance. Accordingly, for questionnaire-derived categorical variables in Supplementary Table S1, percentages were calculated using variable-specific available denominators.

Protein expression values were imputed using the miceforest package in Python. Proteins missing in more than 30% of participants were excluded as predictors for imputing each protein. A single dataset was imputed with up to five iterations, while all the other parameters remained at their default settings. NPX values were scaled from 0 to 1 and centred on the median after analysis [34].

In the proteomic analysis cohort, MetS status was determined within each of the five imputed covariate datasets and consolidated for each participant through majority voting. The probability score was calculated as the mean value across imputations. Migraine labels were sourced from the first imputed dataset, as outcome variables were not imputed.

### 4.5. sJSD-Based Proteomic Divergence Analysis

To identify proteomic signatures that preceded disease onset, a nested case–control design stratified by MetS status was employed. All analyses were conducted separately within the MetS and non-MetS (NoMetS) strata to describe within-stratum proteomic variability patterns; the NoMetS stratum served as a parallel within-stratum reference. Within each stratum, propensity score matching was applied to balance baseline covariates and to define a fixed reference pool [37] (details in Supplementary Figure S3). Incident migraine cases were subsequently grouped into five-time windows based on time-to-event quintiles, which represented the intervals from baseline to migraine diagnosis.

Proteomic signatures were quantified using single-sample Jensen–Shannon divergence (sJSD) [16]. For each stratum, protein expression distributions in cases were compared with those in the reference pool by fitting Gaussian distributions to reference NPX values for each protein, transforming both reference and case NPX values into cumulative probabilities using the normal cumulative distribution function, and calculating the Jensen–Shannon divergence across all measured proteins. An inconsistency index (ICI) was derived as the mean divergence, where higher scores indicate greater deviation from the reference proteomic state.

Differences across time-to-event windows were assessed using pairwise permutation tests (5000 iterations) between the peak window and the others, with multiple-comparison control using Benjamini-Hochberg and Holm methods. The null distribution of the window score was estimated via a 10,000-iteration resampling procedure. Stability was evaluated using 1000-iteration bootstraps on protein subsets and case samples, and peak frequency across windows was tested by chi-square goodness-of-fit. Additional sensitivity analyses examined consistency across different matching specifications and case compositions in each stratum.

### 4.6. Network Proximity Analysis

Disease-associated pathways were identified through overrepresentation analysis using Fisher’s exact test (one-sided, *p* ≤ 0.05) applied to disease–gene associations from the Open Targets Platform (v25.09) [38]. Network-based proximity between disease and pathway genes was quantified as the average shortest-path distance within the STRING protein-protein interaction (PPI) network (v12.0) [39,40]. Statistical significance was determined by comparing observed distances against a null distribution generated through degree-preserving randomization (1000 iterations). Pathways with *z* scores ≤ −2.0 were considered significantly proximal to each disease. Pathways showing significant proximity to both MetS and migraine were designated as shared pathways. Pathway annotations were obtained from Reactome through the Molecular Signatures Database (MSigDB; v2025.1) [41,42].

Pathways shared between MetS and migraine were evaluated through Pearson correlation and linear regression analyses of proximity profiles. Statistical significance was assessed against degree-preserving randomized networks to control for network topology effects. Each pathway was classified as either “Known” or “Novel” based on structured pathway-level literature review. Pathways with published pathway-level associations with MetS, migraine, or a core MetS component were designated “Known”, whereas pathways for which no such pathway-level evidence was identified were designated “Novel”. Here, “Novel” refers to pathways that were previously underrecognized at the pathway level in the context of MetS and migraine, rather than entirely unprecedented biological mechanisms. These shared pathways provided a disease-anchored candidate set for subsequent directed diffusion analysis, ensuring that signal propagation was restricted to routes jointly proximal to both disease modules rather than the entire interactome.

### 4.7. Mediator Subnetwork Identification and Directional Characterization

To identify signalling pathways linking MetS and migraine, Tied Diffusion Through Interacting Events (TieDIE) [20] was applied to the Superpathway Directed Signalling Network (v2.0), which integrates curated directed signalling relationships from multiple pathway resources and is well suited for directionality inference. Disease-associated genes from the Open Targets Platform were used as the source set (MetS) and target set (migraine), with genes filtered to those present in the network. Initial heat values were diffused across the network using a matrix exponentiation–based heat diffusion kernel. Forwards diffusion propagated heat from MetS genes along directed edges, whereas reverse diffusion propagated heat from migraine genes through transposed edges, effectively running the algorithm in the opposite direction. Because diffusion involves continuous heat decay, nodes located closer to the source in the forwards direction display stronger red heat, whereas nodes closer to the target in the reverse direction display stronger blue heat. Nodes exhibiting high heat from both diffusion processes (above the per-direction 75th percentile) were extracted as the mediator subnetwork, representing genes positioned along putative molecular paths connecting MetS and migraine. The 75th percentile cut-off was chosen as a compromise between network sparsity and coverage, and robustness to this choice was evaluated by repeated analyses at the 70th and 80th percentile thresholds.

Pathway enrichment within the mediator subnetwork was evaluated using Fisher’s exact test for the 50 shared pathways identified above. For each pathway, directionality was calculated as the mean MetS-direction heat minus the mean migraine-direction heat across mediator genes. Positive values indicate relative proximity to the MetS source, whereas negative values indicate relative proximity to the migraine target. Directional balance was quantified using a distance-to-diagonal metric, defined as |red_heat − blue_heat|/(red_heat + blue_heat). Bridge score was calculated as the product of red and blue heat. Statistical significance was assessed using the Mann-Whitney U test and generalized linear models that included pathway size and node degree as covariates. Robustness was further evaluated by varying the heat thresholds (70th, 75th, and 80th percentiles) and false discovery rate (FDR) cut-offs (0.01, 0.05, and 0.10).

### 4.8. Drug–Pathway Prioritization and Target Contextualization

Candidate drugs for the direction-consistent novel pathways identified in the directed diffusion analysis were prioritized using ProXimal Pathway Enrichment Analysis (PxEA) [43,44]. PxEA evaluates the topological proximity between drug targets and pathway genes within the PPI network and calculates enrichment statistics similar to gene set enrichment analysis. Statistical significance was determined by permutation testing (1000 iterations), and multiple testing was controlled using the Benjamini-Hochberg method. Before PxEA, drugs were filtered for indications related to MetS or migraine.

Drug target information was retrieved from DrugBank (version 5.1.10) [45]. The overlap between candidate pathway-mapped drug targets and the top 500 dynamic network biomarker proteins identified in the Olink proteomic dataset was examined. For these overlapping proteins, cross-tissue expression validation was performed using data from the Human Protein Atlas [46] and the GTEx Consortium [47]. Associations between protein expression and MetS status were evaluated using analysis of covariance (ANCOVA), adjusted for age and sex.

Effect sizes are reported as Cohen’s *d* values.

### 4.9. Statistical Analysis

All the statistical analyses were performed in R (version 4.2.1) and Python (version 3.9.13). Associations between MetS and incident migraine were examined using Cox proportional hazards regression implemented in the survival package. Within the Cox regression and other statistical models described in this section, R functions were used with their default parameter settings unless explicitly stated otherwise. Three sequential models with increasing levels of covariate adjustment were specified. Model 1 included age, sex, and ethnicity. Model 2 was additionally adjusted for socioeconomic indicators (income, education, and the Townsend deprivation index [tertiles]). Model 3 further incorporated lifestyle factors (smoking status, alcohol consumption, sleep duration, and physical activity). The proportional hazards assumption was verified using Schoenfeld residuals.

Cox models were separately fitted for each of the multiply imputed datasets. Estimates were pooled using Rubin’s rules to combine within- and between-imputation variance, resulting in overall hazard ratios (HRs), standard errors, and 95% confidence intervals (CIs). Dose–response relationships were assessed by modelling the number of MetS components (0–4) as both categorical and continuous exposures. Linear trend tests were conducted across the ordered categories. The independent associations of individual MetS components (central obesity, hypertension, hyperglycaemia, elevated triglycerides, and reduced HDL cholesterol [HDL-C]) were evaluated using mutually adjusted Cox models that included all five components simultaneously. Subgroup analyses were stratified by age, sex, ethnicity, socioeconomic status, and lifestyle factors. Interaction *p* values were obtained by comparing Cox models with and without multiplicative interaction terms for each subgroup variable using a likelihood-ratio chi-square test; *p* values from each imputed dataset were then combined using Fisher’s method.

*p* values less than 0.05 were considered to indicate statistical significance; interaction *p* values were Bonferroni-adjusted across subgroups to correct for multiple testing across the subgroup comparisons. Additional methodological details are provided in the Supplementary Methods.

## 5. Conclusions

This study shifts the MetS–migraine comorbidity framework from static risk assessment towards a diagnosis-stratified view of proteomic divergence and cross-system network organisation. We identified a diagnosis-anchored window with elevated proteomic divergence (4.71–6.76 years before migraine diagnosis) and linked this window to 11 direction-consistent novel pathways and seven candidate bridge proteins spanning metabolic, endothelial, and neural compartments. By integrating divergence-based proteomic analysis, directed diffusion, and drug–pathway proximity, this work provides a tractable computational framework for prioritizing mechanistic and pharmacological hypotheses at the peripheral–central disease interface. Collectively, these findings support prospective evaluation of the T2 window, the identified bridge proteins, and the associated pathway architecture in MetS-related migraine.

## Data Availability

Restrictions apply to the availability of these data. Data were obtained from UK Biobank and are available at https://www.ukbiobank.ac.uk/enable-your-research (accessed on 18 September 2025) with the permission of UK Biobank.

## Supplementary Materials

The following supporting information can be downloaded at: https://www.mdpi.com/article/10.3390/ijms27114820/s1.

## Abbreviations

The following abbreviations are used in this manuscript:

FDR	False discovery rate
FGF2	Fibroblast growth factor 2
ICI	Inconsistency index
IKBKG	Inhibitor of nuclear factor kappa-B kinase regulatory subunit gamma
LUBAC	Linear ubiquitin chain assembly complex
MetS	Metabolic syndrome
MFGE8	Milk fat globule-EGF factor 8 protein
MSigDB	Molecular Signatures Database
NF-κB	Nuclear factor kappa-light-chain-enhancer of activated B cells
NoMetS	Non-metabolic syndrome
NPX	Normalized protein expression
NRGN	Neurogranin
PPI	Protein–protein interaction
PxEA	ProXimal Pathway Enrichment Analysis
sJSD	Single-sample Jensen–Shannon divergence
SRC	Proto-oncogene tyrosine-protein kinase Src
STAT5B	Signal transducer and activator of transcription 5B
TieDIE	Tied Diffusion Through Interacting Events
UKB-PPP	UK Biobank Pharma Proteomics Project
VAV3	Vav guanine nucleotide exchange factor 3
