# Supplementary material for: Integrating Divergence-Based Proteomic Analysis and Directed Network Diffusion to Characterize Diagnosis-Anchored Molecular Variability at the Metabolic Syndrome–Migraine Interface
Source: Int J Mol Sci. 2026 May 27;27(11):4820. doi: 10.3390/ijms27114820 (PMC13257391; doi:10.3390/ijms27114820)
Supplement: Supplementary file 1 [file ijms-27-04820-s001.zip › ijms-4288229-supplementary.pdf]

Supplementary Materials: Integrating Divergence-Based Proteomic Analysis and Directed Network Diffusion to Characterize Diagnosis-Anchored Molecular Variability at the Metabolic Syndrome–Migraine Interface

Bei Wang [1], Yulin Li [1], Yixing Liu [2,*] and Dongran Han [1,2,*]

1. Supplementary Methods

1.1. Single-Sample Jensen–Shannon Divergence (sJSD) Statistical Testing

Propensity score matching was performed using full matching on the logit of the propensity score with caliper = 0.2 SD, exact matching on sex, and estimand = ATT (Average Treatment Effect on Treated), implemented via the MatchIt R package [1]. The matched control set served as the fixed reference pool for all subsequent time-window comparisons. Statistical significance of within-stratum proteomic variability patterns was assessed using a four-tier strategy: (1) window-level ICI differences were evaluated within each stratum separately, with Holm correction applied to the four non-peak windows (T1 and T3–T5), while the peak window T2 was reported using its raw p value; (2) positional specificity of the peak window was evaluated by pairwise permutation tests with 5,000 iterations comparing T2 against T1 and T3–T5, with Benjamini–Hochberg false discovery rate correction across the four comparisons; (3) signal strength was assessed using a 10,000-iteration resampling procedure to generate the null distribution of the T2 score and by neighbor-window permutation tests comparing T2 versus T1 and T2 versus T3, with Holm family-wise error rate control across the two comparisons; (4) stability was assessed by bootstrap resampling with 1,000 iterations on protein subsets and on case samples.

1.2. Additional robustness analyses for the T2-centered proteomic variability

Individual-level normalized ICI was calculated for each incident migraine case to summarize the participant-level values underlying the window-level ICI estimates. For participant i, this value was defined as the average of the participant's protein-level sJSD values across all measured proteins:

$$\mathrm{ICI}_i = \frac{1}{p}\sum_{j=1}^{p} \mathrm{sJSD}_{ij}$$

where p denotes the number of measured proteins. Individual-level ICI values were grouped by diagnosis-anchored window and MetS status, and their distributions were summarized.

A targeted leave-one-out analysis was conducted for the MetS T2 window to evaluate whether the T2 peak was driven by any single participant. Each of the 28 MetS T2 cases was removed one at a time, and the T2 ICI was recalculated using the same fixed MetS reference pool. After each exclusion, the recalculated T2 value was compared with the ICI values from the other four MetS windows. The influence of each omitted participant on the T2 peak was quantified using the per-case mean T2 ICI, calculated as the recalculated T2 ICI divided by the number of remaining cases.

Sensitivity to the number of top-ranked proteins included in the analysis was assessed using prespecified cutoffs of K = 50, 100, 200, 500, 1000, and all 2923 proteins. Proteins were ranked by their T2 gene-level ICI contribution within the MetS group, and window-level ICI values were recalculated for each cutoff. For each cutoff, the T2 ICI was compared with

the highest ICI among the four non-T2 windows to determine whether T2 remained the peak window.

1.3. Network Data Sources

Superpathway v2.0 directed signalling network was used for Tied Diffusion Through Interacting Events (TieDIE) diffusion analysis [2]. STRING protein-protein interaction data (v12.0, medium confidence, combined score ≥ 0.4) was used for network proximity analysis and druggability assessment with degree-matched randomization [3].

1.4. TieDIE Implementation

Mediator subnetwork was defined as nodes with both MetS-direction (forward/red) and migraine-direction (reverse/blue) diffusion heat exceeding the 75th percentile (sensitivity analyses: 70th and 80th percentiles). Bridge score was calculated as the product of red and blue heat. Directional balance was quantified as $|\text{red\_heat} - \text{blue\_heat}|/(\text{red\_heat} + \text{blue\_heat})$. Known versus Novel pathway comparisons used Mann–Whitney U tests with generalized linear model adjustment for pathway size and node degree.

1.5. Pathway Evidence Annotation

Shared pathways were classified as Known or Novel based on structured pathway-level literature review. A pathway was classified as Known if at least one published study reported a pathway-level association with metabolic syndrome, migraine, or a core metabolic syndrome component (including obesity, insulin resistance, type 2 diabetes, dyslipidaemia, or NAFLD/NASH). Pathways for which no such pathway-level evidence was identified through database-assisted retrieval and manual review were classified as Novel. Here, "Novel" denotes pathways that were previously underrecognized at the pathway level rather than entirely unprecedented biology. This annotation was used for interpretive stratification of shared pathways and did not affect the network proximity or directed diffusion calculations.

1.6. ProXimal Pathway Enrichment Analysis (PxEA) Druggability Assessment

Drugs from DrugBank v5.1.10 (small molecules only) were filtered to include those with known indications for metabolic syndrome or migraine, based on DrugBank indication field keyword matching. MetS-related keywords: diabetes, diabetic, hyperglycemia, insulin resistance, hypertension, hypertensive, hyperlipidemia, dyslipidemia, hypercholesterolemia, hypertriglyceridemia, obesity, metabolic syndrome, cardiovascular, atherosclerosis. Migraine-related keywords: migraine, headache, cephalalgia. This filtering enabled assessment of whether direction-consistent Novel pathways are already targeted by established drugs, providing evidence of druggability.

1.7. Tissue Expression Validation

Expression of candidate bridge proteins (SRC, IKBKG, FGF2, MFGE8, NRGN, STAT5B, VAV3) was validated using tissue and brain expression data from the Human Protein Atlas, including GTEx-derived tissue TPM (transcripts per million) values and single-cell expression profiles [4]. Tissues were categorized as brain-related (13 regions) or metabolism-related (8 tissues). Expression values were $\log_2$-transformed, z-score standardized within genes across tissues, and visualized by hierarchical clustering.

1.8. Top 500 Divergence-Associated Protein Selection

Proteins were ranked by their per-protein cumulative sJSD contribution in the T2 window, obtained by summing the Jensen–Shannon divergence values for each protein across all T2 case samples relative to the reference distribution. The top 500 proteins with

the highest T2-window cumulative sJSD scores were designated as the high-divergence set. To support this cutoff, a one-sided Mann–Whitney U test was used to compare T2-window cumulative sJSD scores of the top 500 proteins against those of proteins ranked 501–1000.

1.9. Olink Cohort Validation

Plasma expression of seven candidate bridge proteins was evaluated in the Olink Explore 3072 baseline dataset. Although the underlying dataset contained four baseline-defined groups (Healthy, MetS_only, Migraine_only, MetS_Migraine), the primary between-group validation reported in the main text was restricted to participants without baseline migraine, comparing the MetS_only and Healthy groups [5]. Quality control required ≥60% non-missing NPX values per protein at baseline (instance_0); because the input dataset did not include assay-specific LOD flags, detection rate was defined as the proportion of non-NA measurements. Group differences for this primary comparison were evaluated using ANCOVA (analysis of covariance) adjusted for age and sex, with Benjamini–Hochberg correction applied across the seven proteins. Effect sizes were calculated as Cohen's *d* and Cliff's delta.

1.10. Software and Reproducibility

Analyses were performed in R and Python. Key R packages: survival, MatchIt, missRanger, sva, effsize, ggplot2. Key Python packages: numpy, pandas, scipy, NetworkX, statsmodels, miceforest. Fixed random seeds were used where applicable to improve reproducibility. All statistical tests were two-sided except for: (1) over-representation analyses (Fisher's exact test, alternative = "greater"); (2) bridge specificity comparisons (one-sided Mann–Whitney U tests: Novel > Known for bridge scores, Novel < Known for distance-to-diagonal, with Bonferroni correction); and (3) TOP500 divergence ranking validation (one-sided Mann–Whitney U test: TOP500 > Rank 501–1000). Multiple comparison adjustments are specified in figure legends and table footnotes.

2. Supplementary Tables

Table S1. Baseline characteristics of the Cox analysis cohort according to metabolic syndrome status.

Variable	Total (*n* = 452,471)	NoMetS (*n* = 284,935)	MetS (*n* = 167,536)	*p* value
Age (years)	56.64 (8.11)	55.69 (8.21)	58.27 (7.68)	<0.001
**Sex**				<0.001
Female	237,692 (52.5)	164,249 (57.6)	73,443 (43.8)	
Male	214,779 (47.5)	120,686 (42.4)	94,093 (56.2)	
**Ethnicity**				<0.001
Asian	10,946 (2.4)	6,498 (2.3)	4,448 (2.7)	
Black	7,693 (1.7)	5,400 (1.9)	2,293 (1.4)	
Mixed	2,669 (0.6)	1,896 (0.7)	773 (0.5)	
Other	4,260 (0.9)	2,701 (0.9)	1,559 (0.9)	
White	426,903 (94.3)	268,440 (94.2)	158,463 (94.6)	
**Income**				<0.001
£18k–30k	102,132 (25.1)	61,990 (24.2)	40,142 (26.8)	
£31k–51k	104,227 (25.7)	68,729 (26.8)	35,498 (23.7)	
£52k–100k	78,334 (19.3)	54,505 (21.3)	23,829 (15.9)	
>£100k	20,261 (5.0)	14,942 (5.8)	5,319 (3.5)	
<£18k	101,245 (24.9)	56,159 (21.9)	45,086 (30.1)	
**Education**				<0.001

Continued on next page

Table S1 – continued from previous page

**Variable**	**Total (n = 452,471)**	**NoMetS (n = 284,935)**	**MetS (n = 167,536)**	p **value**
A levels	48,815 (11.0)	32,264 (11.5)	16,551 (10.1)	
CSEs	17,055 (3.8)	10,793 (3.9)	6,262 (3.8)	
None	79,865 (18.0)	41,814 (15.0)	38,051 (23.3)	
NVQ/HND/HNC	57,361 (12.9)	34,226 (12.3)	23,135 (14.1)	
O levels/GCSEs	58,379 (13.2)	36,807 (13.2)	21,572 (13.2)	
Professional	39,842 (9.0)	24,180 (8.7)	15,662 (9.6)	
University	141,673 (32.0)	99,270 (35.5)	42,403 (25.9)	
**Social deprivation**				<0.001
Least deprived	150,824 (33.3)	98,270 (34.5)	52,554 (31.4)	
Medium	150,824 (33.3)	95,713 (33.6)	55,111 (32.9)	
Most deprived	150,823 (33.3)	90,952 (31.9)	59,871 (35.7)	
**Smoking**				<0.001
Current	48,834 (10.8)	29,770 (10.5)	19,064 (11.4)	
Never	245,623 (54.5)	163,958 (57.7)	81,665 (49.0)	
Previous	156,094 (34.6)	90,193 (31.8)	65,901 (39.5)	
**Alcohol**				<0.001
Daily	93,006 (20.6)	60,847 (21.4)	32,159 (19.2)	
Never	35,828 (7.9)	20,071 (7.1)	15,757 (9.4)	
1–2 times/week	117,506 (26.0)	74,675 (26.2)	42,831 (25.6)	
1–3 times/month	49,089 (10.9)	29,696 (10.4)	19,393 (11.6)	
Special occasions	51,323 (11.4)	29,299 (10.3)	22,024 (13.2)	
3–4 times/week	105,147 (23.3)	70,019 (24.6)	35,128 (21.0)	
**Sleep duration**				<0.001
Long (>9 h)	8,267 (1.8)	3,946 (1.4)	4,321 (2.6)	
Normal (6–9 h)	415,665 (92.7)	264,724 (93.6)	150,941 (91.0)	
Short (<6 h)	24,678 (5.5)	14,130 (5.0)	10,548 (6.4)	
**Physical activity**				<0.001
1–1.5 h	108,542 (24.0)	66,645 (23.4)	41,897 (25.0)	
1.5–2 h	27,940 (6.2)	19,016 (6.7)	8,924 (5.3)	
15–30 min	114,596 (25.3)	68,361 (24.0)	46,235 (27.6)	
2–3 h	20,789 (4.6)	14,276 (5.0)	6,513 (3.9)	
30–60 min	114,320 (25.3)	74,715 (26.2)	39,605 (23.6)	
<15 min	6,094 (1.3)	3,408 (1.2)	2,686 (1.6)	
>3 h	60,071 (13.3)	38,447 (13.5)	21,624 (12.9)	

Note: Continuous variables are presented as mean (standard deviation), and categorical variables as number (percentage). p values are derived from two-sample t tests for continuous variables and χ^2 tests for categorical variables comparing participants with versus without metabolic syndrome, based on the first imputed dataset used for the main Cox regression analysis. For questionnaire-derived categorical variables, percentages were calculated among participants with available data for that variable; therefore, category counts may not sum to the column totals. Missing or "prefer not to answer" responses retained during post-imputation covariate recoding were as follows: Income, 46,272 overall (28,610 in NoMetS; 17,662 in MetS); Education, 9,481 overall (5,581 in NoMetS; 3,900 in MetS); Smoking, 1,920 overall (1,014 in NoMetS; 906 in MetS); Alcohol, 572 overall (328 in NoMetS; 244 in MetS); Sleep duration, 3,861 overall (2,135 in NoMetS; 1,726 in MetS); and Physical activity, 119 overall (67 in NoMetS; 52 in MetS). For categorical variables, the p value is reported once in the header row of each variable and left blank for individual category rows; χ^2 tests were performed on observed non-missing categories.

Table S2. Heterogeneity of MetS–migraine association across subgroups.

Subgroup Variable	Interaction p value	Bonferroni-corrected p value
Age (<50 vs ≥50 years)	1.65×10^{-6}	1.65×10^{-5}
Sex (Male vs Female)	0.988	1.000
Ethnicity	0.761	1.000
Income	0.008	0.078
Education	0.791	1.000
Social deprivation	0.967	1.000
Smoking status	0.855	1.000
Alcohol consumption	0.911	1.000
Sleep duration	0.792	1.000
Physical activity	0.420	1.000

Note: Interaction p values were obtained by comparing Cox proportional hazards models with and without multiplicative interaction terms between MetS and each subgroup variable using a likelihood-ratio chi-square test. Interaction p values from each imputed dataset were combined using Fisher's method. Bonferroni correction was applied across the 10 subgroup comparisons; the corresponding significance threshold for the uncorrected interaction p values was 0.005 (0.05/10). Bonferroni-corrected p values are presented in the third column.

Table S3. Summary statistics of inconsistency index (ICI) across time windows.

Window	Time to diagnosis (years)	MetS ICI	MetS SE	MetS 95% CI	NoMetS ICI	NoMetS SE	NoMetS 95% CI
T1	[2.04, 4.71)	0.602	0.004	0.594–0.610	0.937	0.004	0.929–0.944
T2	[4.71, 6.76)	0.715	0.005	0.705–0.725	0.970	0.004	0.962–0.978
T3	[6.76, 9.42)	0.605	0.004	0.597–0.614	0.954	0.004	0.946–0.961
T4	[9.42, 11.90)	0.613	0.004	0.605–0.622	0.971	0.004	0.963–0.979
T5	[11.90, 14.41]	0.657	0.005	0.648–0.666	0.922	0.004	0.915–0.929

Note: ICI, inconsistency index; SE, standard error; CI, confidence interval. Standard errors were estimated via bootstrap resampling (1,000 iterations). Each window contained 28 MetS and 42 NoMetS incident migraine cases, matched to reference pools of 15,579 (MetS) and 25,976 (NoMetS) controls. Time to diagnosis windows were defined as half-open intervals [2.04, 4.71), [4.71, 6.76), [6.76, 9.42), [9.42, 11.90), and [11.90, 14.41], based on quintiles of time to event (years).

Table S4. Pairwise comparisons of ICI values between T2 and other time windows in the MetS and NoMetS cohorts.

Stratum	Comparison	ICI difference	p (permutation)	q (BH-adjusted)
MetS	T2 vs T1	0.113	0.024	0.048
MetS	T2 vs T3	0.110	0.027	0.048
MetS	T2 vs T4	0.102	0.036	0.048
MetS	T2 vs T5	0.058	0.151	0.151
NoMetS	T2 vs T1	0.033	0.290	0.507
NoMetS	T2 vs T3	0.016	0.397	0.507
NoMetS	T2 vs T4	−0.001	0.507	0.507
NoMetS	T2 vs T5	0.048	0.214	0.507

Note: ICI, inconsistency index; BH, Benjamini–Hochberg. p values were derived from pairwise permutation tests (5,000 iterations) comparing T2 against each other window within each metabolic stratum. The Benjamini–Hochberg procedure was applied separately within each stratum across the four T2-versus-other-window comparisons to control the false discovery rate at $\alpha = 0.05$. In the MetS cohort, T2 ICI significantly exceeded T1, T3, and T4 (all $q < 0.05$), while the comparison with T5 did not reach significance ($q = 0.151$) despite an 8.8% elevation. In the NoMetS cohort, none of the T2-versus-other-window comparisons reached statistical significance (all $q = 0.507$).

Table S5. Summary statistics of the null distribution from the resampling test for the MetS T2 peak.

Metric	Value	Description
Observed T2 ICI	0.7153	Observed ICI value in T2 window from real data
Null mean	0.6376	Mean ICI from 10,000 random resampling iterations
Null SD	0.0349	Standard deviation of null distribution
Percentile 95th	0.6972	95th percentile of null distribution
Percentile 99th	0.7231	99th percentile of null distribution
p value	0.018	Proportion of null values exceeding observed T2
Z-score	2.22	Standardized deviation from null mean

Note: The null distribution was generated by randomly sampling 28 MetS cases for the T2 window position across 10,000 iterations, while keeping other windows fixed. The observed T2 ICI (0.7153) exceeded 98.2% of the null distribution (p = 0.018), indicating that the T2 peak is unlikely to have arisen by chance. Z-score represents the standardized deviation of the observed value from the null mean. Full-precision values are reported in this table to support resampling statistics; the main text and Tables S3 and S7 present rounded values (0.715) for readability.

Table S6. Bootstrap stability validation for the MetS T2 peak.

Bootstrap type	Comparison	Observed difference	Bootstrap 95% CI	Proportion positive	T2 peak frequency
Protein subset	T2 vs T1	0.113	[0.109, 0.118]	100.00%	100%
Protein subset	T2 vs T3	0.110	[0.106, 0.115]	100.00%	100%
Case resampling	T2 vs T1	0.113	[0.012, 0.213]	98.70%	85%
Case resampling	T2 vs T3	0.110	[0.009, 0.210]	98.50%	85%

Note: Bootstrap resampling (1,000 iterations) was performed using two strategies to assess the stability of the T2 peak. Protein subset bootstrap resampled proteins with replacement while keeping case samples fixed, testing whether the peak is robust to protein selection. Case resampling bootstrap resampled case samples with replacement while keeping all proteins, testing whether the peak is robust to case composition.

Table S7. Parameter robustness validation of the MetS T2 peak across different settings.

Parameter setting	T2 ICI	T1 ICI	T3 ICI	Peak window	Δ vs T1	Δ vs T3
Primary analysis (global reference pool)	0.715	0.602	0.605	T2	18.8%	18.2%
Sensitivity 1 (local reference pool)	0.775	0.735	0.635	T2	5.4%	22.0%
Sensitivity 2 (top 10% proteins)	0.264	0.179	0.226	T2	47.7%	16.9%

Note: ICI, inconsistency index; Δ, percentage change calculated from full-precision ICI values before rounding. Displayed ICI values are rounded to three decimals and therefore may not exactly reproduce the reported percentage changes. Sensitivity analyses were performed under different parameter settings while maintaining the same temporal window definitions and case selection.

Table S8. Window-specific case numbers and individual-level ICI summary.

Stratum	Window	*n*	Mean	SD	Median	P25	P75	P5–P95
MetS	T1	28	0.0215	0.0057	0.0206	0.0178	0.0244	0.0146–0.0322
MetS	T2	28	0.0255	0.0102	0.0216	0.0188	0.0308	0.0163–0.0455
MetS	T3	28	0.0216	0.0061	0.0190	0.0175	0.0234	0.0160–0.0340
MetS	T4	28	0.0219	0.0052	0.0216	0.0182	0.0245	0.0159–0.0285
MetS	T5	28	0.0235	0.0086	0.0204	0.0180	0.0240	0.0157–0.0393
NoMetS	T1	42	0.0223	0.0050	0.0221	0.0181	0.0257	0.0158–0.0294
NoMetS	T2	42	0.0231	0.0080	0.0201	0.0183	0.0245	0.0151–0.0377
NoMetS	T3	42	0.0227	0.0077	0.0199	0.0182	0.0242	0.0160–0.0402
NoMetS	T4	42	0.0231	0.0062	0.0215	0.0186	0.0262	0.0158–0.0363
NoMetS	T5	42	0.0220	0.0055	0.0205	0.0182	0.0238	0.0157–0.0312

Note: ICI, inconsistency index; SD, standard deviation; P5, 5th percentile; P25, 25th percentile; P75, 75th percentile; P95, 95th percentile. Individual-level normalized ICI was calculated for each incident migraine case as the average protein-level sJSD across all measured proteins. Within each window, the sum of the unrounded participant-level values corresponds to the window-level ICI estimate reported in the main sJSD analysis. The T1–T5 windows follow the same diagnosis-anchored intervals reported in Supplementary Table S3.

Table S9. Leave-one-out influence analysis for the MetS T2 window.

LOO metric	Result	Interpretation
Full-sample T2 ICI	0.7153	Window-level ICI for the full MetS T2 sample before leave-one-out analysis.
Full-sample per-case mean T2 ICI	0.02555	Full-sample T2 ICI divided by 28 MetS T2 cases.
MetS T2 cases	28	Number of incident migraine cases in the MetS T2 window.
LOO iterations	28	One MetS T2 case was removed in each iteration.
Iterations retaining T2 as peak	27/28 (96.4%)	Iterations in which T2 remained the highest-ICI MetS window after recalculation.
Iterations shifting peak away from T2	1/28 (3.6%)	In the single shifted iteration, the peak moved marginally to T5 (recalculated T2 ICI = 0.65687; T5 ICI = 0.65690).
LOO per-case mean T2 ICI	0.02555 [0.02458–0.02591]	Mean and 2.5th–97.5th percentile interval across leave-one-out iterations.
Maximum |Δ| per-case mean T2 ICI	0.00122	Largest absolute change from the full-sample per-case mean T2 ICI across leave-one-out iterations.
Median |Δ| per-case mean T2 ICI	0.00024	Median absolute change from the full-sample per-case mean T2 ICI across leave-one-out iterations.

Note: ICI, inconsistency index; LOO, leave-one-out. The analysis was targeted to the MetS T2 window. The same fixed MetS reference pool was used for recalculating T2 ICI after each omitted case. Per-case mean T2 ICI was calculated as the recalculated window-level T2 ICI divided by the number of remaining cases and was used to quantify the influence of each omitted participant.

Table S10. Top-K cutoff sensitivity analysis for the MetS T2 peak.

K	*n*	Peak	T2 rank	T2 ICI (top *K*)	T2 mean/case (top *K*)	ΔICI vs. best non-T2
50	28	T2	1	1.397	0.0499	0.603
100	28	T2	1	1.350	0.0482	0.554
200	28	T2	1	1.282	0.0458	0.490
500	28	T2	1	1.139	0.0407	0.362
1000	28	T2	1	1.002	0.0358	0.251
2923	28	T2	1	0.715	0.0255	0.058

Note: ICI, inconsistency index. Proteins were ranked by their T2 gene-level ICI contribution within the MetS group. For each prespecified cutoff *K*, window-level ICI values were recalculated within the selected top-*K* protein subset. The primary sensitivity criterion was whether T2 remained the peak window across these prespecified cutoffs. ΔICI vs. best non-T2 denotes the absolute ICI difference between T2 and the highest-ICI non-T2 window. The full-protein cutoff $K = 2923$ includes all measured proteins and reproduces the main all-protein MetS T2 ICI.

Table S11. Complete information for 50 shared pathways (rows 1–15).

Pathway abbreviation	Expanded label	Gene set name	Database	MetS z-score	Migraine z-score	Novelty	TOP500 overlap
IRAK1 RECRUITS IKK cpx	Irak1 Recruits Ikk Complex	REACTOME_IRAK1_RECRUITS_IKK_COMPLEX	Reactome	−353.59	−48.75	Known	1
JNK C JUN KINASES phos AND act med BY ACTIVATED HUMAN TAK1	Jnk C Jun Kinases Phosphorylation And Act Mediated By Activated Human Tak1	REACTOME_JNK_C_JUN_KINASES_ PHOSPHORYLATION_AND_ACTIVATION_MEDIATED_ BY_ACTIVATED_HUMAN_TAK1	Reactome	−353.59	−48.75	Known	1
SLC15A4 TASL dep IRF5 act	Slc15a4 Tasl Dep Irf5 Activation	REACTOME_SLC15A4_TASL_DEPENDENT_IRF5_ ACTIVATION	Reactome	−316.62	−47.15	Known	1
reg OF RUNX1 expr AND act	Reg Of Runx1 Expr And Activation	REACTOME_REGULATION_OF_RUNX1_EXPRESSION_ AND_ACTIVITY	Reactome	−205.20	−36.60	Known	1
ACTIVATED NTRK3 sig THROUGH PI3K	Activated Ntrk3 Signalling Through Pi3k	REACTOME_ACTIVATED_NTRK3_SIGNALS_THROUGH_ PI3K	Reactome	−205.20	−36.60	Known	1
nucl sig BY ERBB4	Nucl Signalling By Erbb4	REACTOME_NUCLEAR_SIGNALING_BY_ERBB4	Reactome	−204.67	−36.20	Known	1
NETRIN 1	Netrin 1	REACTOME_NETRIN_1_SIGNALING	Reactome	−204.67	−36.20	Known	1
ADP sig THROUGH P2Y PURINOCEPTOR 1	Adp Signalling Through P2y Purinoceptor 1	REACTOME_ADP_SIGNALLING_THROUGH_P2Y_ PURINOCEPTOR_1	Reactome	−204.67	−36.20	Known	1
MAP2K AND MAPK act	Map2k And Mapk Activation	REACTOME_MAP2K_AND_MAPK_ACTIVATION	Reactome	−193.97	−37.43	Known	1
VEGFR2 med CELL prolif	Vegfr2 Mediated Cell Proliferation	REACTOME_VEGFR2_MEDIATED_CELL_PROLIFERATION	Reactome	−193.97	−37.43	Known	1
sig BY NTRK3 TRKC	Sig By Ntrk3 Trkc	REACTOME_SIGNALING_BY_NTRK3_TRKC	Reactome	−175.22	−36.36	Known	1
DOWNreg OF ERBB4	Downreg Of Erbb4	REACTOME_DOWNREGULATION_OF_ERBB4_ SIGNALING	Reactome	−175.22	−39.09	Known	1
GRB2 SOS PROVIDES link TO MAPK sig FOR integrS	Grb2 Sos Provides Link To Mapk Signalling For Integrins	REACTOME_GRB2_SOS_PROVIDES_LINKAGE_TO_ MAPK_SIGNALING_FOR_INTEGRINS	Reactome	−162.65	−36.36	Known	1
IKK cpx RECRUITMENT med BY RIP1	Ikk Complex Recruitment Mediated By Rip1	REACTOME_IKK_COMPLEX_RECRUITMENT_ MEDIATED_BY_RIP1	Reactome	−113.44	−25.27	Known	2
TICAM1 RIP1 med IKK cpx RECRUITMENT	Ticam1 Rip1 Mediated Ikk Complex Recruitment	REACTOME_TICAM1_RIP1_MEDIATED_IKK_COMPLEX_ RECRUITMENT	Reactome	−98.14	−24.52	Known	2

Table S11 – continued from previous page (rows 16–30)

Pathway abbreviation	Expanded label	Gene set name	Database	MetS z-score	Migraine z-score	Novelty	TOP500 overlap
sig BY ERBB4	Sig By Erbb4	REACTOME_SIGNALING_BY_ERBB4	Reactome	−71.79	−27.02	Known	2
GAB1 SIGNALOSOME	Gab1 Signalosome	REACTOME_GAB1_SIGNALOSOME	Reactome	−71.79	−25.81	Known	2
NFKB sig AND ARTD FAMILY MEMBERS	Nfkb Signalling And Artd Family Members	WP_NFKB_SIGNALING_AND_ARTD_FAMILY_MEMBERS	WikiPathways	−357.99	−47.19	Novel	1
reg OF NF KAPPA B	Reg Of Nf Kappa B	REACTOME_REGULATION_OF_NF_KAPPA_B_SIGNALING	Reactome	−357.99	−47.19	Novel	1
reg OF TNF NFKB sig path LUBAC med lin ubiq	Reg Of Tnf Nfkb Signalling Path Lubac Mediated Lin Ubiq	KEGG_MEDICUS_REFERENCE_REGULATION_OF_TNF_NFKB_SIGNALING_PATHWAY_LUBAC_MEDIATED_LINEAR_UBIQUITINATION	KEGG	−353.59	−48.75	Novel	1
DNA IRdmg AND cell resp VIA ATR	Dna Irdmg And Cell Resp Via Atr	WP_DNA_IRDAMAGE_AND_CELLULAR_RESPONSE_VIA_ATR	WikiPathways	−352.60	−46.91	Novel	1
pregn X R	Pregn X R	WP_PREGNANE_X_RECEPTOR_PATHWAY	WikiPathways	−205.20	−36.60	Novel	1
ER nongenom	Er Non-Genomic	PID_ER_NONGENOMIC_PATHWAY	PID (NCI)	−205.20	−36.60	Novel	1
reg OF commis AXON pathfind BY SLIT AND ROBO	Reg Of Commis Axon Pathfind By Slit And Robo	REACTOME_REGULATION_OF_COMMISSURAL_AXON_PATHFINDING_BY_SLIT_AND_ROBO	Reactome	−205.20	−36.60	Novel	1
SYNDECAN 3	Syndecan 3	PID_SYNDECAN_3_PATHWAY	PID (NCI)	−205.20	−36.60	Novel	1
E2 ER RAS ERK	E2 Er Ras Erk	KEGG_MEDICUS_REFERENCE_E2_ER_RAS_ERK_SIGNALING_PATHWAY	KEGG	−205.20	−36.60	Novel	1
CA2 PYK2 RAS ERK	Ca2 Pyk2 Ras Erk	KEGG_MEDICUS_REFERENCE_CA2_PYK2_RAS_ERK_SIGNALING_PATHWAY	KEGG	−205.20	−36.60	Novel	1
path shig IPAC TO ACTIN	Pathway: Shig Ipac To Actin	KEGG_MEDICUS_PATHOGEN_SHIGELLA_IPAC_TO_ACTIN_SIGNALING_PATHWAY	KEGG	−205.20	−36.60	Novel	1
CHRNA7 E2F	Chrna7 E2f	KEGG_MEDICUS_REFERENCE_CHRNA7_E2F_SIGNALING_PATHWAY	KEGG	−205.20	−36.60	Novel	1
P130CAS link TO MAPK sig FOR integrS	P130cas Link To Mapk Signalling For Integrins	REACTOME_P130CAS_LINKAGE_TO_MAPK_SIGNALING_FOR_INTEGRINS	Reactome	−204.67	−36.20	Novel	1

Table S11 – continued from previous page (rows 31–50)

Pathway abbreviation	Expanded label	Gene set name	Database	MetS z-score	Migraine z-score	Novelty	TOP500 overlap
path HCMV GH TO ITGA B RHOA	Pathway: Hcmv Gh To Itga B Rhoa	KEGG_MEDICUS_PATHOGEN_HCMV_GH_TO_ITGA_B_RHOA_SIGNALING_PATHWAY	KEGG	−204.67	−36.20	Novel	1
NETRIN med repuls	Netrin Mediated Repuls	REACTOME_NETRIN_MEDIATED_REPULSION_SIGNALS	Reactome	−204.67	−36.20	Novel	1
path HBV HBX TO RAS ERK	Pathway: Hbv Hbx To Ras Erk	KEGG_MEDICUS_PATHOGEN_HBV_HBX_TO_RAS_ERK_SIGNALING_PATHWAY	KEGG	−204.67	−36.20	Novel	1
ENV fact E2 TO RAS ERK	Env Fact E2 To Ras Erk	KEGG_MEDICUS_ENV_FACTOR_E2_TO_RAS_ERK_SIGNALING_PATHWAY	KEGG	−204.67	−36.20	Novel	1
ENV fact NNK NNN TO CHRNA7 E2F	Env Fact Nnk Nnn To Chrna7 E2f	KEGG_MEDICUS_ENV_FACTOR_NNK_NNN_TO_CHRNA7_E2F_SIGNALING_PATHWAY	KEGG	−204.67	−36.20	Novel	1
AMPLIFICATION AND EXPANSION OF ONCOGENIC pathS AS METASTATIC TRAITS	Amplification And Expansion Of Oncogenic Paths As Metastatic Traits	WP_AMPLIFICATION_AND_EXPANSION_OF_ONCOGENIC_PATHWAYS_AS_METASTATIC_TRAITS	WikiPathways	−193.97	−37.43	Novel	1
INLA med entr OF list monocyt INTO host cells	Inla Mediated Entr Of List Monocyt Into Host Cells	REACTOME_INLA_MEDIATED_ENTRY_OF_LISTERIA_MONOCYTOGENES_INTO_HOST_CELLS	Reactome	−193.97	−37.43	Novel	1
ALPHA 6 BETA 4	Alpha 6 Beta 4	WP_ALPHA_6_BETA_4_SIGNALING	WikiPathways	−193.97	−37.43	Novel	1
ERBB2 ERBB3	Erbb2 Erbb3	PID_ERBB2_ERBB3_PATHWAY	PID (NCI)	−193.97	−37.43	Novel	1
AR nongenom	Ar Non-Genomic	PID_AR_NONGENOMIC_PATHWAY	PID (NCI)	−193.97	−37.43	Novel	1
P4 PR RAS ERK	P4 Pr Ras Erk	KEGG_MEDICUS_REFERENCE_P4_PR_RAS_ERK_SIGNALING_PATHWAY	KEGG	−193.97	−37.43	Novel	1
LONG TERM POTENTIATION	Long Term Potentiation	REACTOME_LONG_TERM_POTENTIATION	Reactome	−193.97	−37.43	Novel	1
SIGNAL AMPLIFICATION	Signal Amplification	REACTOME_SIGNAL_AMPLIFICATION	Reactome	−175.22	−36.36	Novel	1
DCC med attr	Dcc Mediated Attr	REACTOME_DCC_MEDIATED_ATTRACTIVE_SIGNALING	Reactome	−175.22	−39.09	Novel	1
ROBO4 AND VEGF	Robo4 And Vegf	WP_ROBO4_AND_VEGF_SIGNALING_CROSSTALK	WikiPathways	−162.65	−39.09	Novel	1
list monocyt entr INTO host cells	List Monocyt Entr Into Host Cells	REACTOME_LISTERIA_MONOCYTOGENES_ENTRY_INTO_HOST_CELLS	Reactome	−77.47	−24.59	Novel	2
EPHA2 FWD	Epha2 Fwd	PID_EPHA2_FWD_PATHWAY	PID (NCI)	−32.15	−19.88	Novel	2
EPO R	Epo R	WP_EPO_RECEPTOR_SIGNALING	WikiPathways	−31.66	−18.71	Novel	2
AVB3 integr	Avb3 Integr	PID_AVB3_INTEGRIN_PATHWAY	PID (NCI)	−9.72	−16.87	Novel	3
EGF EGFR ACTIN	Egf Egfr Actin	KEGG_MEDICUS_REFERENCE_EGF_EGFR_ACTIN_SIGNALING_PATHWAY	KEGG	−2.36	−19.15	Novel	3

Note: Pathway abbreviation: Short labels displayed in Fig. 4C. Expanded label: Reader-friendly expanded descriptors corresponding to the pathway abbreviations shown in Fig. 4C, provided for readability rather than as database-standard pathway titles. Gene set name: Collection-specific gene set name used in the pathway proximity analysis; prefixes indicate the source pathway collection. Database: Source pathway collection inferred from the gene set name prefix (Reactome, KEGG, WikiPathways, or PID). MetS/Migraine z-score: Network proximity z-scores from degree-preserving randomization (1,000 iterations); more negative values indicate closer proximity. Novelty: Classification based on pathway-level disease evidence identified through structured literature review, as described in the Supplementary Methods. TOP500 overlap: Number of ICI-contributing proteins from the T2 window overlapping with this pathway (range 1–3).

Table S12. Complete TieDIE directed diffusion results for 16 pathways significantly enriched in the mediator subnetwork.

Pathway name	Classification	Network genes	Mediator genes	Fold enrichment	p value	FDR (BH)	MetS heat	Migraine heat	Directionality
Slc15a4 Tasl Dependent Irf5 Activation	Known	4	3	5.70	8.17E−03	3.14E−02	0.304	0.306	−0.002
Ikk Complex Recruitment Mediated By Rip1	Known	19	12	4.80	5.06E−07	2.53E−05	0.362	0.428	−0.066
Ticam1 Rip1 Mediated Ikk Complex Recruitment	Known	16	9	4.28	5.38E−05	6.73E−04	0.367	0.462	−0.095
Grb2 Sos Provides Linkage To Mapk Signalling For Integrins	Known	15	8	4.06	2.35E−04	1.68E−03	0.357	0.479	−0.121
Map2k And Mapk Activation	Known	30	14	3.55	7.68E−06	1.92E−04	0.387	0.536	−0.149
Regulation Of Tnf Nfkb Signalling Pathway Lubac Mediated Linear Ubiquitination	Novel	3	3	7.60	2.27E−03	1.13E−02	0.326	0.316	0.010
Env Factor E2 To Ras Erk Signalling Pathway	Novel	13	7	4.09	5.51E−04	3.06E−03	0.437	0.624	−0.188
P130cas Linkage To Mapk Signalling For Integrins	Novel	15	8	4.06	2.35E−04	1.68E−03	0.357	0.479	−0.121
Nfkb Signalling And Artd Family Members	Novel	19	9	3.60	3.01E−04	1.88E−03	0.369	0.447	−0.077
Epo Receptor Signalling	Novel	24	11	3.49	9.18E−05	9.18E−04	0.354	0.428	−0.074
E2 Er Ras Erk Signalling Pathway	Novel	14	6	3.26	5.95E−03	2.48E−02	0.452	0.675	−0.223
Regulation Of Nf Kappa B Signalling	Novel	12	5	3.17	1.38E−02	4.33E−02	0.305	0.304	0.001
Ca2 Pyk2 Ras Erk Signalling Pathway	Novel	12	5	3.17	1.38E−02	4.33E−02	0.431	0.624	−0.193
Pregnane X Receptor Pathway	Novel	15	6	3.04	8.83E−03	3.15E−02	0.405	0.532	−0.127
Long Term Potentiation	Novel	18	7	2.96	5.64E−03	2.48E−02	0.527	0.866	−0.338
Avb3 Integrin Pathway	Novel	63	21	2.54	2.81E−05	4.68E−04	0.390	0.463	−0.072

Note: Network genes and mediator genes denote pathway genes present in the Superpathway network and in the TieDIE mediator subnetwork, respectively. Enrichment was tested by one-sided Fisher's exact test with Benjamini–Hochberg FDR correction ($q < 0.05$). MetS heat and Migraine heat are the mean forward and reverse diffusion heat across mediator genes within each pathway. Directionality was calculated as MetS heat − Migraine heat; positive values indicate relative proximity to the MetS source, whereas negative values indicate relative proximity to the migraine target.

Table S13. Characteristics of seven candidate bridge proteins identified at the MetS–migraine intersection.

Gene symbol	Novel pathways (n)	Pathway names	Cohen's d	q-value	Brain z-score	Metabolic z-score
MFGE8	1	AVB3 Integrin	0.56	0.00e+00	−0.82	0.52
IKBKG	3	NF-κB Signalling; TNF-LUBAC-NFκB; NFκB+ARTD	0.20	3.35×10^{-97}	−0.55	0.76
STAT5B	1	EPO Receptor Signalling	0.20	9.17×10^{-92}	−0.38	0.27
NRGN	1	Long-Term Potentiation	0.14	4.16×10^{-50}	1.12	−0.34
FGF2	1	AVB3 Integrin	0.14	2.26×10^{-43}	0.82	−0.49
VAV3	1	AVB3 Integrin	0.13	2.94×10^{-40}	−0.51	−0.47
SRC	6	E2-RAS-ERK; CA2-PYK2-RAS-ERK; E2-ER-RAS-ERK; P130CAS-MAPK; EPO Receptor Signalling; PXR	0.08	4.89×10^{-17}	−0.93	−0.55

Note: Cohen's d: Effect size for NPX difference between MetS-only individuals (n = 17,739) and healthy controls (n = 29,420) in UK Biobank. Calculated as (mean_MetS − mean_Healthy) / pooled_SD. q-value: False discovery rate (FDR)-adjusted p value from Benjamini–Hochberg correction; all $q < 0.05$ indicates significant elevation in MetS. Brain z-score: Mean z-score of protein expression across brain tissues (GTEx); positive values indicate brain enrichment. Metabolic z-score: Mean z-score of protein expression across metabolic tissues (liver, adipose, skeletal muscle, pancreas); positive values indicate metabolic tissue enrichment.

Table S14. Candidate drugs identified by PxEA for MetS–migraine Novel pathways.

Drug name	Indication category	Enrichment score	p value	FDR	Targeted pathway names
Carvedilol	MetS	104.4	<0.001	<0.001	E2-RAS-ERK; CA2-PYK2-RAS-ERK; E2-ER-RAS-ERK; Long-Term Potentiation; P130CAS-MAPK; EPO Receptor Signalling; PXR
Valproic acid	Migraine	101.1	<0.001	<0.001	E2-RAS-ERK; CA2-PYK2-RAS-ERK; E2-ER-RAS-ERK; TNF-LUBAC-NFκB; Long-Term Potentiation; P130CAS-MAPK; NF-κB Signalling; EPO Receptor Signalling; NFκB+ARTD; PXR
Terazosin	MetS	75.1	<0.001	<0.001	TNF-LUBAC-NFκB; AVB3 Integrin; NF-κB Signalling; NFκB+ARTD

Note: p value: Statistical significance of enrichment score from permutation test (n = 1,000 permutations). p values < 0.001 indicate 0 of 1,000 permutations exceeded the observed enrichment score. FDR: False discovery rate from Benjamini–Hochberg correction; threshold for significance = 0.05. Two additional drugs, indomethacin and acetylsalicylic acid, were significant by PxEA (FDR < 0.05) but did not directly target any of the 11 Novel pathways (0/11); therefore, this table lists only the three direct-targeting drugs shown in Fig. 6e. Abbreviated pathway labels in Tables S13–S14 correspond to the full pathway names reported in Table S12 and the short labels shown in Fig. 4C.

3. Supplementary Figures

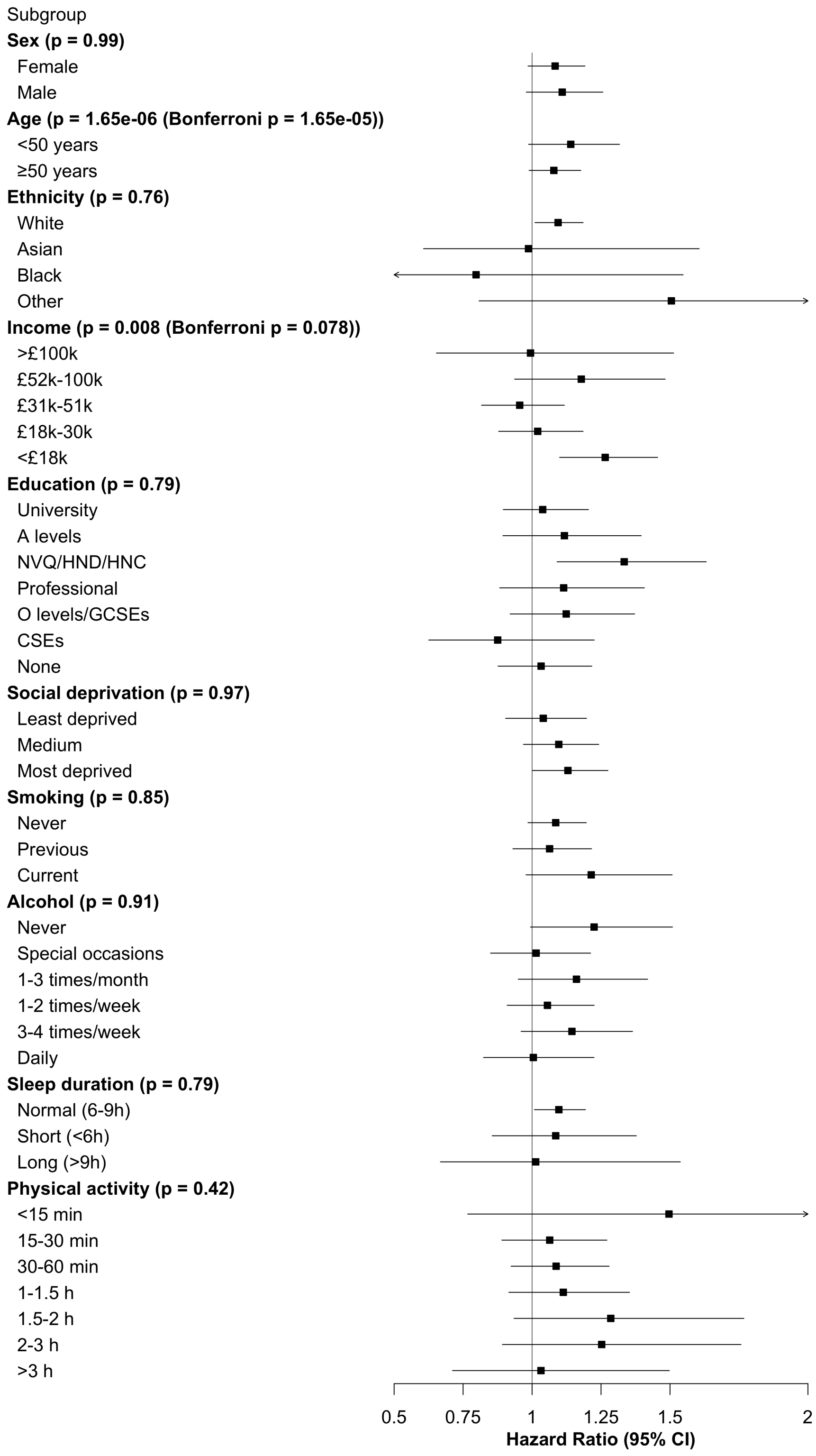

Figure S1. Subgroup analysis of metabolic syndrome and incident migraine. Hazard ratios (squares) with 95% confidence intervals (horizontal lines) from Cox proportional hazards models. *p* values are for MetS × subgroup interaction assessed by likelihood-ratio chi-square test; Bonferroni-adjusted *p* values are shown in parentheses.

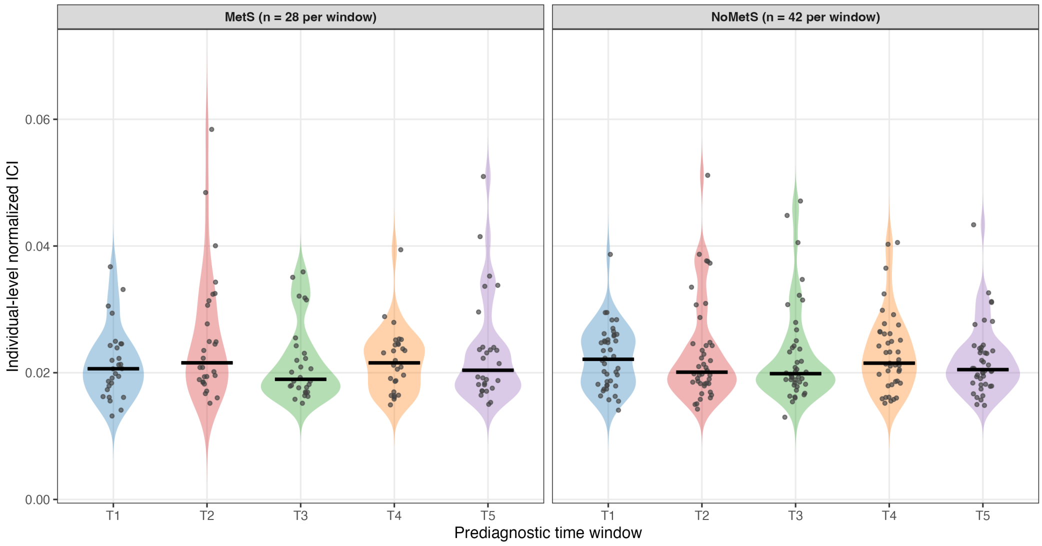

Figure S2. Individual-level ICI distributions across diagnosis-anchored windows and MetS strata. Individual-level normalized ICI was calculated for each incident migraine case as the average protein-level sJSD across all measured proteins. Violin plots show the descriptive distributions of these values across T1–T5 windows in the MetS and NoMetS groups; each point represents one incident migraine case, and black horizontal bars indicate median values. Within each window, the sum of the unrounded participant-level values corresponds to the window-level ICI estimate reported in the main sJSD analysis. The two panels are displayed on a common y-axis scale; the MetS panel includes 28 cases per window, and the NoMetS panel includes 42 cases per window. ICI, inconsistency index; MetS, metabolic syndrome; sJSD, single-sample Jensen–Shannon divergence.

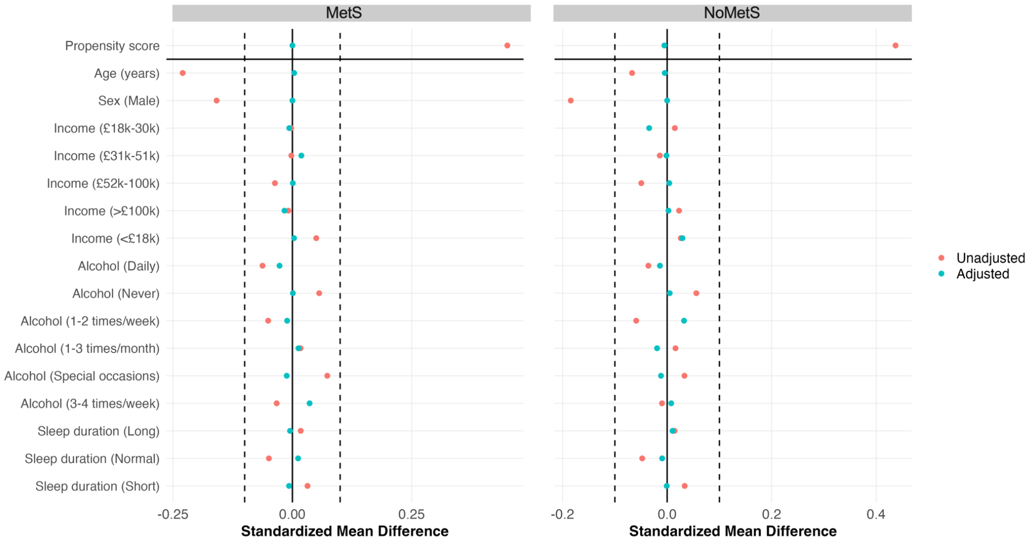

Figure S3. Propensity score matching balance diagnostics (Love plots). (**a**) MetS stratum. (**b**) NoMetS stratum. Points show standardized mean differences (SMD) for each covariate before matching (Unadjusted, red) and after full matching (Adjusted, blue). Vertical dashed lines at $x = \pm 0.10$ indicate the balance threshold; the solid vertical line marks $x = 0$. Matching used full matching on the logit of the propensity score with exact matching on sex, a caliper of 0.2 SD on logit(PS), and the ATT estimand. All covariates achieved $|\text{SMD}| \leq 0.10$ after matching. Abbreviations: MetS, metabolic syndrome; SMD, standardized mean difference; PS, propensity score.

References

1. Ho, D.; Imai, K.; King, G.; Stuart, E. MatchIt: nonparametric preprocessing for parametric causal inference. *J. Stat. Softw.* **2011**, *42*, 1–28. https://doi.org/10.18637/jss.v042.i08.

2. Paull, E.; Carlin, D.; Niepel, M.; Sorger, P.; Haussler, D.; Stuart, J. Discovering causal pathways linking genomic events to transcriptional states using Tied Diffusion Through Interacting Events (TieDIE). *Bioinformatics* **2013**, *29*, 2757–2764. https://doi.org/10.1093/bioinformatics/btt471.
3. Szklarczyk, D.; Kirsch, R.; Koutrouli, M.; Nastou, K.; Mehryary, F.; Hachilif, R.; Gable, A.; Fang, T.; Doncheva, N.; Pyysalo, S.; et al. The STRING database in 2023: protein-protein association networks and functional enrichment analyses for any sequenced genome of interest. *Nucleic Acids Res.* **2023**, *51*, D638–D646. https://doi.org/10.1093/nar/gkac1000.
4. GTEx Consortium. The GTEx consortium atlas of genetic regulatory effects across human tissues. *Science* **2020**, *369*, 1318–1330. https://doi.org/10.1126/science.aaz1776.
5. Dhindsa, R.; Burren, O.; Sun, B.; Prins, B.; Matelska, D.; Wheeler, E.; Bernal-Rubio, J.; Campos, L.; Tan, A.; González-Galarza, F. Rare variant associations with plasma protein levels in the UK Biobank. *Nature* **2023**, *622*, 339–347. https://doi.org/10.1038/s41586-023-06547-x.

Disclaimer/Publisher's Note: The statements, opinions and data contained in all publications are solely those of the individual author(s) and contributor(s) and not of MDPI and/or the editor(s). MDPI and/or the editor(s) disclaim responsibility for any injury to people or property resulting from any ideas, methods, instructions or products referred to in the content.
